# Signals from the deep: Spatial and temporal acoustic occurrence of beaked whales off western Ireland

**DOI:** 10.1371/journal.pone.0199431

**Published:** 2018-06-21

**Authors:** Katie Kowarski, Julien Delarue, Bruce Martin, Joanne O’Brien, Rossa Meade, Oliver Ó. Cadhla, Simon Berrow

**Affiliations:** 1 JASCO Applied Sciences, Dartmouth, Nova Scotia, Canada; 2 Marine and Freshwater Research Centre, Galway-Mayo Institute of Technology, Galway, Ireland; 3 National Parks & Wildlife Service, Department of Culture, Heritage and the Gaeltacht, Dublin, Ireland; Evergreen State College, UNITED STATES

## Abstract

Little is known of the spatio-temporal occurrence of beaked whales off western Ireland, limiting the ability of Regulators to implement appropriate management and conservation measures. To address this knowledge gap, static acoustic monitoring was carried out using eight fixed bottom-mounted autonomous acoustic recorders: four from May to December 2015 on Ireland’s northern slope and four from March to November 2016 on the western and southern slopes. Recorders ran for 205 to 230 days, resulting in 4.09 TB of data sampled at 250 kHz which could capture beaked whale acoustic signals. Zero-crossing-based automated detectors identified beaked whale clicks. A sample of detections was manually validated to evaluate and optimize detector performance. Analysis confirmed the occurrence of Sowerby’s and Cuvier’s beaked whales and Northern bottlenose whales. Northern bottlenose whale clicks occurred in late summer and autumn, but were too few to allow further analysis. Cuvier’s and Sowerby’s clicks occurred at all stations throughout the monitoring period. There was a significant effect of month and station (latitude) on the mean daily number of click detections for both species. Cuvier’s clicks were more abundant at lower latitudes while Sowerby’s were greater at higher latitudes, particularly in the spring, suggesting a spatial segregation between species, possibly driven by prey preference. Cuvier’s occurrence increased in late autumn 2015 off northwest Porcupine Bank, a region of higher relative occurrence for each species. Seismic airgun shots, with daily sound exposure levels as high as 175 dB re 1 μPa^2^·s, did not appear to impact the mean daily number of Cuvier’s or Sowerby’s beaked whale click detections. This work provides insight into the significance of Irish waters for beaked whales and highlights the importance of using acoustics for beaked whale monitoring.

## Introduction

The conservation and management of marine mammal populations is inherently constrained by the quantity and quality of available information on the ecology and distribution of the species of interest [1–3]. Collecting reliable data on some cetacean species that are highly mobile, deep-diving, wide ranging, and rare is particularly challenging. This problem could result in inadequate conservation measures [4, 5] or a lack of compliance with international directives [6]. Such issues are particularly topical for species belonging to the Ziphiidae family, known as beaked whales.

Beaked whales are among the most diverse but least known cetaceans globally, with comparatively little information available on their distribution, ecology and population structure [7, 8]. They occur worldwide, typically in offshore pelagic habitats where they show a preference for continental slope waters, deep ocean troughs, and canyons [9–11]. Their deep oceanic habitat and elusive behaviour including long, deep dives followed by short surface intervals have made these species difficult to study using traditional line-transect methods that are constrained by weather conditions [2, 4]. Ziphiid movements and distribution are likely driven by that of their prey. For example, Giorli et al. [12] hypothesized that Ziphiids move between areas to optimize their access to prey in Hawaii. Indeed, beaked whale foraging behaviour differs across species, oceans, habitats, time of day, and season [2, 12–15]. Globally, beaked whales are considered Data Deficient, except for the Cuvier’s beaked whale (*Ziphius cavirostris*) and Southern bottlenose whale (*Hyperoodon planifrons*) which have been categorized as species of Least Concern [16]. Observations and research in recent decades has shown beaked whales to be sensitive to several human maritime activities, particularly the use of mid-frequency military sonar which has been linked to behavioural changes, temporary area avoidance, and fatal mass stranding events [17–26]. It has further been shown that shipping may affect beaked whale diving and foraging behaviour and intense sound sources from seismic surveys are also of concern [27]. Even small changes in the dive cycle of beaked whales, caused by anthropogenic disturbance, could cause decompression sickness since long dive times result in relatively high nitrogen concentration in their tissues [27–30], though such an acute outcome was not observed during controlled exposure studies (e.g. [24, 31, 32]). As information on beaked whale responses to human activities in the ocean grows, it is increasingly necessary to improve our baseline understanding of their distribution, habitat use and ecology [2].

Ireland’s Exclusive Economic Zone holds significant energy resources that are expected to contribute to the development of the Irish economy and to Ireland’s energy security. Nearshore areas are currently being investigated for wave, wind, and tidal energy installations. The western part of the Irish continental shelf and down the continental slope, an area known as the Atlantic Margin, is thought to offer considerable potential reserves of oil and gas. The Atlantic Margin’s continental slope is topographically diverse, being cut by several troughs and canyons, and it has been described as one of the more biologically productive regions of the North-East Atlantic, making it potentially suitable habitat for many cetaceans, including Ziphiids [6, 33, 34].

Five beaked whale species have been reported in Irish waters thus far. Cuvier’s beaked whales were observed during surveys that included the Atlantic Margin [4, 33, 35–38]. This species is the most frequently stranded beaked whale in Ireland, leaving some to speculate that Cuvier’s may breed in the region [3, 34]. Northern bottlenose whales (*Hyperoodon ampullatus*) have stranded on 50 occasions [3, 39] and have been infrequently sighted [4, 33, 38]. Sowerby’s beaked whales (*Mesoplodon bidens*) have occasionally been observed offshore [4, 33, 38] and the few strandings have not shown any seasonality [3]. There has only been one sighting thought to be True’s beaked whale (*Mesoplodon mirus*) along the Atlantic Margin [33]. Finally, Gervais’ beaked whales (*Mesoplodon europaeus*) are known in Ireland from a single stranding [40]. In the UK, Blainville’s beaked whales (*M*. *densirostris*) have stranded on two occasions [41–43] and therefore may also occur in Irish waters. Offshore Atlantic surveys in effective weather conditions have largely been limited to summer months with all studies concluding that research on a wider spatio-temporal scale is required to understand the distribution and movements of these cryptic animals [4, 6, 33, 38].

All cetacean species in Irish waters are protected under national and international legislation [44, 45]. To effectively achieve this mandate, knowledge gaps regarding the spatial and temporal use of areas of interest for oil and gas exploration or other industrial practices by marine mammals must be investigated and addressed. In this regard, a comprehensive acoustic project, ObSERVE Acoustic, was conceived and commissioned by Ireland’s Department of Communications, Climate Action and Environment, and the Department of Culture, Heritage and the Gaeltacht to provide robust data with which to inform conservation and management in the Atlantic Margin by assessing the importance of these shelf edge habitats for beaked whales and other cetaceans.

We used passive acoustic monitoring (PAM) techniques to monitor for Ziphiids on an unprecedented scale. Smaller scale PAM projects have previously successfully recorded beaked whale acoustics in the area [46, 47], but were rarely able to confidently identify the species concerned. As a monitoring technique, PAM offers several benefits over conventional visual survey methods. For example, data can be collected continuously in a cost-effective manner in deep, offshore, remote waters regardless of light and weather conditions. Detection of cetaceans is, however, dependent on their sound production. Marine mammals rely on sound for many vital functions including navigation, foraging, and breeding [48, 49]. Although the acoustic signals of many beaked whale species remain unknown or poorly described, among North Atlantic species the clicks of Northern Bottlenose whale [50–52], Cuvier’s [53, 54], Sowerby’s [55], and Gervais’ beaked whales [53, 56] have been described. Notably, the click characteristics are unique for each species which allows for reliable acoustic identification. In this study, we assessed the spatial and temporal distribution of acoustically active beaked whale species in the Atlantic Margin over the course of two years using static acoustic recorders.

## Methods

### Data collection

**Fig 1:**
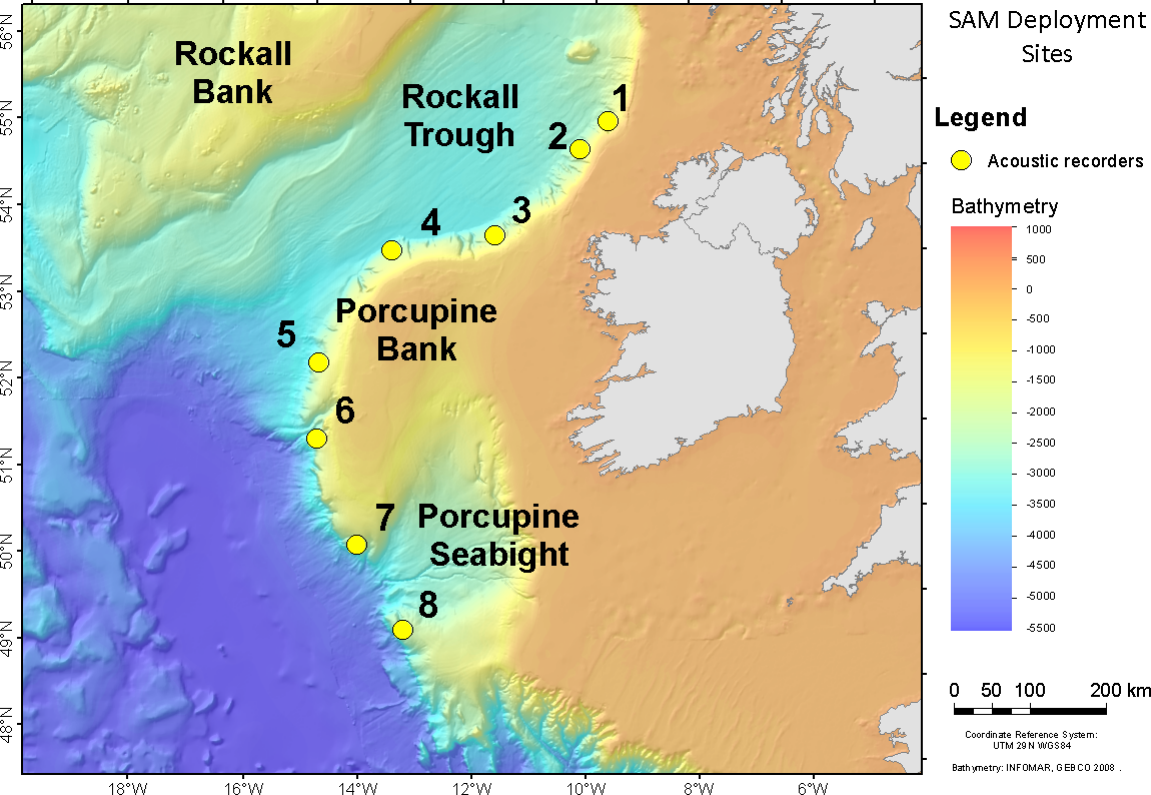
Acoustic monitoring stations using AMARs in the Atlantic Margin study area off western Ireland during 2015 (stations 1–4) and 2016 (stations 3 and 5–8).

Acoustic data were collected using Autonomous Multichannel Acoustic Recorders (AMARs; JASCO Applied Sciences) suspended approximately 15 m above the seafloor at four locations in 2015 (stations 1–4) and at five locations in 2016 (stations 3 and 5–8; Fig 1 and Table 1). Data collection occurred over four recording periods: May to Aug 2015, Aug to Dec 2015, Mar to July/Aug 2016, and Jul/Aug to Oct/Nov 2016 (Table 1). In 2015, each AMAR was fitted with an HTI−99-HF omnidirectional hydrophone (High Tech Inc., −164 dB re 1 V/μPa sensitivity). In 2016, M36-V35-100 omnidirectional hydrophones (GeoSpectrum, −165 dB re 1 V/μPa sensitivity) were used. Recorders sampled on 8 min duty cycles in 2015 and 14.5 to 15 min duty cycles in 2016 (Table 2). Low sampling rates (32 and 2 kHz) were used to record lower frequency marine mammal vocalizations. The low frequency channels had 24-bit resolution with a spectral noise floor of 29 dB re 1 μPa^2^/Hz and a nominal ceiling of 165 dB re 1 μPa. High sampling rate (250 kHz) channels were used to record high frequency odontocete clicks, including those of beaked whales that are described here. The high frequency channels had 16-bit resolution with a spectral noise floor of 35 dB re 1 μPa^2^/Hz and a nominal ceiling of 171 dB re 1 μPa. Acoustic data were stored on internal solid-state flash memory for post-retrieval processing. Instrument retrieval was achieved via an acoustic release.

**Table 1:** The location, depth, and operation period of AMARs deployed off western Ireland at eight stations.

Station	Latitude (°N)	Longitude (°W)	Depth (m)	Start day	End day	Year	Total no. of recording days
**1**	55.6302	−9.7302	1600	08 May	22 Aug	2015	214
	55.6323	−9.7252	1620	29 Aug	13 Dec	2015	
**2**	55.3018	-10.3084	1995	08 May	22 Aug	2015	214
	55.3011	−10.3037	1971	29 Aug	13 Dec	2015	
**3**	54.2513	−11.9940	1850	30 Aug	14 Dec	2015	205
	54.2502	−11.9926	1770	24 Mar	29 Jun	2016	
**4**	54.0014	−14.0424	1920	07 May	20 Aug	2015	213
	54.0015	−14.0429	1944	30 Aug	14 Dec	2015	
**5**	52.6225	−15.3045	1752	21 Mar	10 Jul	2016	227
	52.6221	-15.3046	1750	10 Jul	02 Nov	2016	
**6**	51.7226	−15.2077	1765	20 Mar	11 Jul	2016	210
	51.7245	-15.2342	1745	11 Jul	15 Oct	2016	
**7**	50.5096	−14.3124	1750	19 Mar	11 Jul	2016	230
	50.5085	-14.3150	1750	11 Jul	03 Nov	2016	
**8**	49.5477	−13.3730	1760	19 Mar	9 Aug	2016	230
	49.5478	-13.3723	1760	09 Aug	03 Nov	2016	

**Table 2:** The duty cycle, including duration at each sampling rate, of AMARs deployed in May—Dec of 2015, March—Jul of 2016, and July—Nov of 2016.

Deployment timeframe	Duty Cycle (min)	Sampling rate (kHz)	Duration (sec)
**May-Dec 2015**	8	32	342
		250	130
		sleep	8
**Mar-Jul 2016**	14.5	2	678
		250	95
		sleep	90
**Jul-Nov 2016**	15	32	680
		250	97
		sleep	90

The 32 and 2 kHz sampling rate channels had 24-bit resolution with a spectral noise floor of 29 dB re 1 μPa^2^/Hz and a nominal ceiling of 165 dB re 1 μPa. The 250 kHz sampling rate channels had 16-bit resolution with a spectral noise floor of 35 dB re 1 μPa^2^/Hz and a nominal ceiling of 171 dB re 1 μPa.

### Automatic detection

Odontocete clicks were identified using JASCO’s custom combined energy-detector and classification algorithm. The software used a Teager-Kaiser energy detector to identify potential clicks in the data and then computed three zero-crossing features of each click: the number of zero-crossings, the median time between zero-crossings, and the median change in time between zero-crossings. These features were compared to an acoustic library of odontocete clicks known to be from beaked whales and each click was classified as the species with the lowest Mahalanobis distance from equivalent library template parameters. From here on, the combined detector-classifier is referred to as the detector or the automated detector and its outputs are called detections.

To determine automated detector performance and verify species occurrence, a selection of acoustic files was visually and aurally reviewed by experienced bio-acoustic analysts using PAMlab (JASCO Applied Sciences). Files selected for manual review were distributed across time of year, time of day, the number of automated detections, and the number of species automatically detected (both beaked whales and other odontocetes). The presence or absence of identifiable signals of every beaked whale species, as well as any other sounds of interest including seismic impulses and naval sonar, was noted for every file reviewed. Beaked whale species were identified based on species-specific click characteristics previously described in the literature [2, 50–56]. Note that the validation process characterizes the ability of the automated detector to identify at least one click in each file and does not discern between files in which all clicks were correctly identified and those in which only a portion of clicks was correctly identified. It does not characterize the performance of the automated detector in identifying individual clicks. This method allowed us to calculate the automated detector performance. We captured the frequency at which automated detectors missed calls (false negatives; FN), correctly identified them (true positives; TP), and incorrectly identified them (false positive; FP) on a ‘per acoustic file’ basis. To optimize our results, we utilized a maximum likelihood estimator that compared manual and automated results to determine the minimum number of automated detections per acoustic file, i.e. the detection threshold, that maximized the “F-score” [57]:

$$F=\frac{(1+\beta^{2})P*R}{(\beta^{2})P+R};P=\frac{TP}{TP+FP};R=\frac{TP}{TP+FN}$$

where the automated detector’s precision (P) is the proportion of detections that are accurate, and recall (R) is the proportion of recorded beaked whale clicks that are automatically detected on a per file basis [58, 59]. To maximize the reliability of our results, a greater emphasis was placed on precision than recall (β = 0.5).

One-min sound files in which the automated detections met or exceeded the minimum detection threshold are presented as species presence/absence per acoustic file in daily and hourly occurrence plots. The mean number of automated detections per day are presented by station and month excluding detections in any acoustic files where the minimum detection threshold was not met. Although the acoustic data at stations 1–4 and 5–8 were recorded in 2015 and 2016, respectively, they are displayed on the same monthly time scale for ease of reading and interpretation.

During manual validation of beaked whale presence, seismic impulses were observed. Therefore, JASCO’s seismic pulse sequence detector [60] was used to identify periods with seismic survey pulses. The software detects energy within pre-defined time frequency bins that is at least three times greater than the median value and then joins adjacent bins thereby creating contours. Contours 0.2–6-seconds in duration with a bandwidth of at least 60 Hz are retained. The software algorithm looks for repeating retained contour events with spacings of 4.8 – 65s, the normal range of seismic pulse periods. Where at least six regularly spaced contours occur, the automated detector signals the occurrence of a seismic pulse sequence. Results from the seismic pulse sequence detector were compared to seismic survey events noted during manual validation of beaked whale clicks and these were found to accurately reflect seismic occurrence.

### Statistical analysis

Statistical analysis was carried out for each beaked whale species detected with high accuracy (F-score > 0.80). First, a multivariate analysis of variance (MANOVA) tested for the difference in the number of mean daily detections across months, stations, and days where seismic signals were present versus absent. Parameters identified by the MANOVA as significant were further explored. The null hypothesis that the mean number of detections per day of each species was constant across the eight stations was tested with non-parametric Kruskal-Wallis tests [61]. Where the null hypothesis was rejected, a Tukey-Kramer multiple comparison test allowed us to determine which stations differed. The same statistical techniques were implemented to test the null hypothesis that, for each species, the mean number of detections per recording day was constant across recording months.

Diel patterns were explored using all days with detections across every station. For every day, each hour containing detections (detection hour) was categorized as occurring in one of four light periods based on nautical time: dawn (sun is 12^o^ below horizon to sunrise), light (sunrise to sunset), dusk (sunset to when the sun is below the horizon by 12^o^), and dark (sun is less than 12^o^ below the horizon). Sunset, sunrise, nautical dusk, and nautical dawn times were acquired from [62]. For every day, the number of detection hours in each light period was divided by the total hours in the associated light period to produce the average detection hours per light period. In this manner, detection occurrence was normalized and the variability in daily light periods over the course of the study was accounted for. To address the daily variation in the number of detection hours, the mean detection hours of each day was subtracted from the average detection hours per light period, resulting in the mean adjusted detection hours. Welch’s ANOVA was used to test the null hypothesis that the mean adjusted detection hours were constant across diel light periods [61]. A Tukey-Kramer multiple comparison test determined if the mean-adjusted hours with detections differed significantly between any diel light periods [63, 64].

## Results

Static acoustic monitoring resulted in 1656.8 days (19.26 TB) of acoustics data, of which 4.09 TB had a sampling rate of 250 kHz and was thus suitable for beaked whale occurrence analysis.

### Beaked whale detector performance

**Fig 2:**
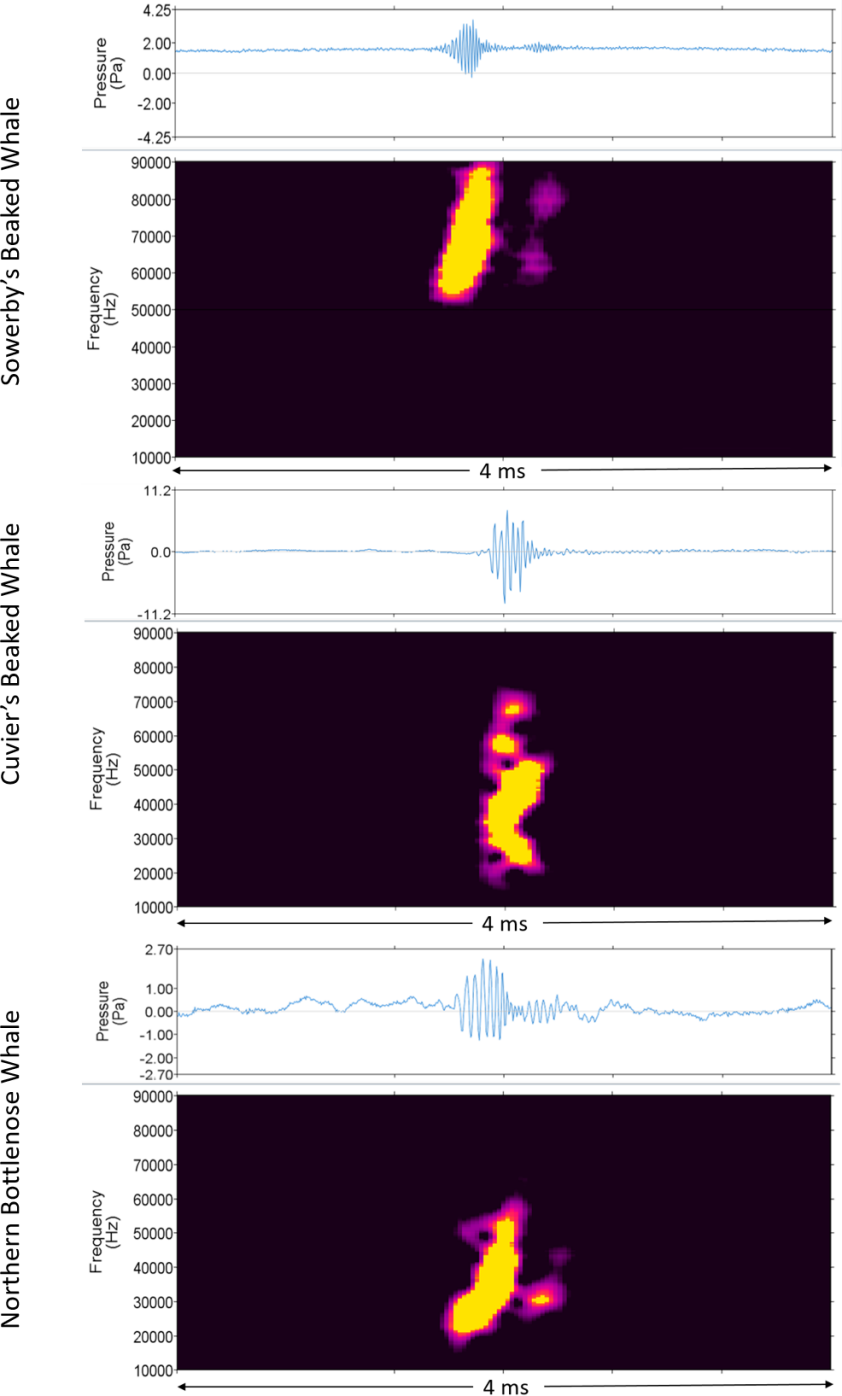
**Waveform (top) and spectrogram (bottom) of beaked whale clicks.** A Sowerby’s beaked whale click recorded at station 1 on 5 Sept 2015 at 14:26, a Cuvier’s beaked whale click recorded at station 3 on 29 Nov 2015 at 17:56, and a northern bottlenose whale click recorded at station 1 on 12 Sept 2015 at 20:10 (right; 512 Hz frequency resolution, 2.6 ms time window, 0.2 ms time step, Hamming window).

A manual review of 2,530 acoustic files (1.07% of recordings or 74.3 hours of data) sampled at 250 kHz confirmed the presence of beaked whales in 780 acoustic files. Cuvier’s and Sowerby’s beaked whales and northern bottlenose whale clicks were observed (Fig 2). Clicks attributed to Cuvier’s beaked whales and northern bottlenose whales were based on previous descriptions of these species by Zimmer et al. [54] and Martin et al. [52], respectively. The click type denoted as Sowerby’s beaked whale is similar in frequency characteristics to the high frequency Sowerby’s beaked whale click described from surface recordings by Cholewiak et al. [55] and it matches the click type identified as Sowerby’s beaked whale by Stanistreet et al. [2]. We did not observe clicks matching any of the lower frequency Sowerby’s beaked whale click types described by Cholewiak et al. [55], potentially reflecting a difference between surface versus deep diving (foraging) click behaviour.

The Cuvier’s beaked whale click classifier had a precision of 0.87 and recall of 0.79 (F-score = 0.86, Table 3). The Sowerby’s beaked whale click classifier performed similarly well, capturing 93% of acoustic files where Sowerby’s occurred, of which 96% truly contained Sowerby’s beaked whale clicks (F-score = 0.95, Table 3). The northern bottlenose whale click classifier required optimization via implementation of a detector threshold. Acoustic files with less than 15 detections were not included resulting in a precision of 1.00, though only 42% of acoustic files where northern bottlenose signals occurred were captured (F-score = 0.78, Table 3).

**Table 3:** Automated detector precision (P), recall (R), and F-score (F) prior (original) and after (optimized) application of detection thresholds for the beaked whale species detected off western Ireland on static acoustic recorders in 2015 and 2016.

Species	P^Original^	R^Original^	F^Original^	Threshold	P^Optimized^	R^Optimized^	F^Optimized^
**Cuvier’s**	0.87	0.79	0.86	1	0.87	0.79	0.86
**Sowerby’s**	0.96	0.93	0.95	1	0.96	0.93	0.95
**Northern bottlenose**	0.11	0.79	0.13	15	1.00	0.42	0.78

### Cuvier’s beaked whales

**Fig 3:**
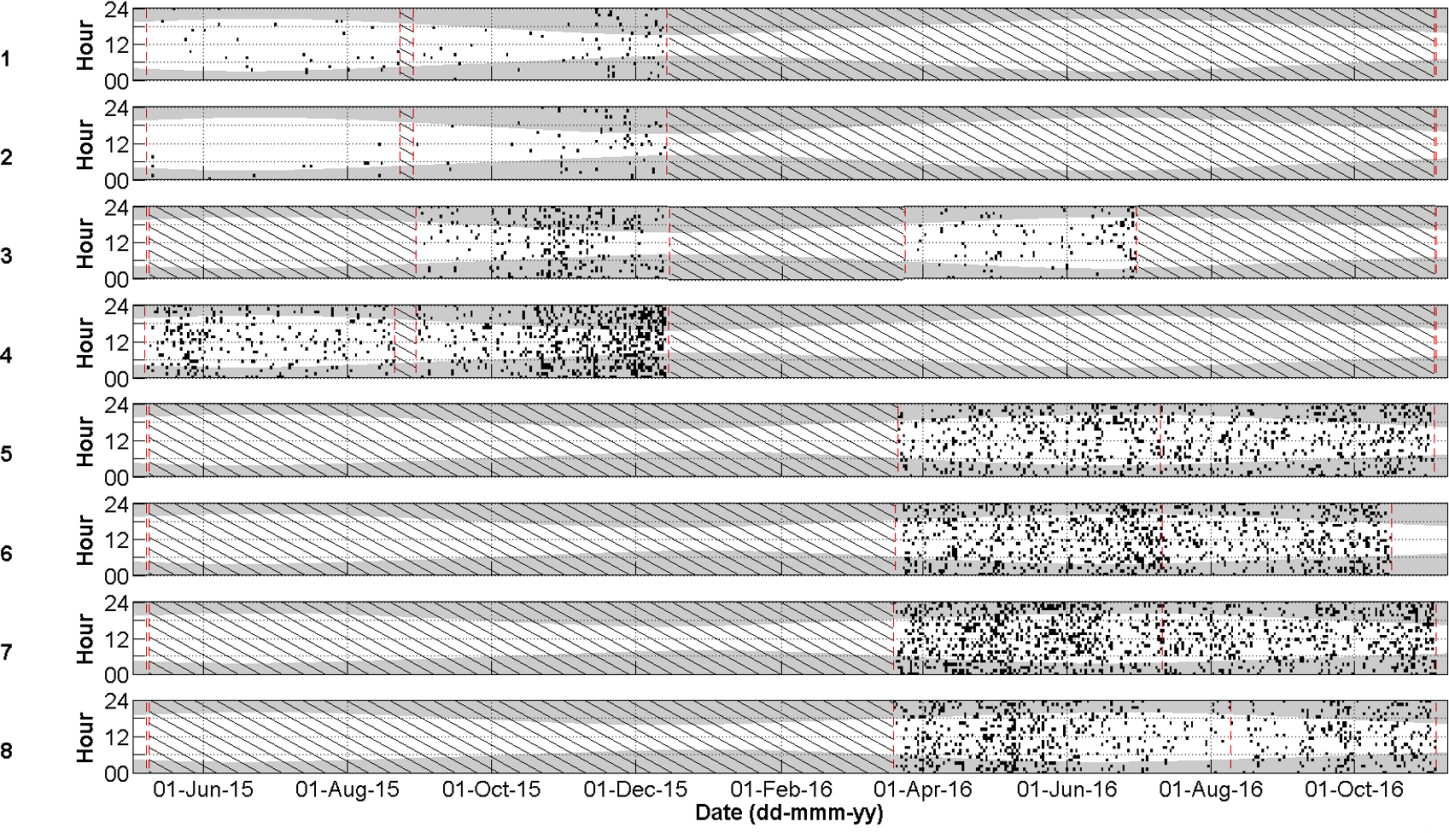
Daily and hourly occurrence of Cuvier’s beaked whale clicks per acoustic file at stations 1–8 represented as individual black dots (P = 0.87, R = 0.79). Shaded areas indicate periods of darkness. The red dashed lines indicate the deployment and retrieval dates. Black diagonal lines indicate periods with no data.

**Fig 4:**
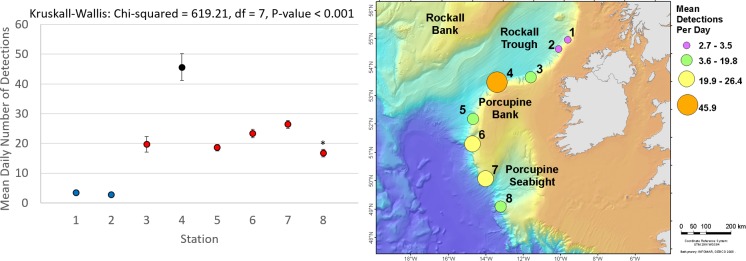
**Mean Cuvier’s beaked whale detections per recording day bubble plot (right) and with standard error bars (left) for stations 1 (SE = ±0.70), 2 (SE = ±0.59), 3 (SE = ±2.61), 4 (SE = ±4.48), 5 (SE = ±1.08), 6 (SE = ±1.38), 7 (SE = ±1.35), and 8 (SE = ±1.26) off western Ireland from May to Dec 2015 (stations 1–4) and Mar to Nov 2016 (stations 3 and 5–8).** Results of Kruskall-Wallis tests are given and results from Tukey-Kramer multiple comparisons tests are represented by coloured means (left) with different colours denoting a statistically significant difference. *Station 8 differs from 7, but not from 3, 5, and 6.

**Fig 5:**
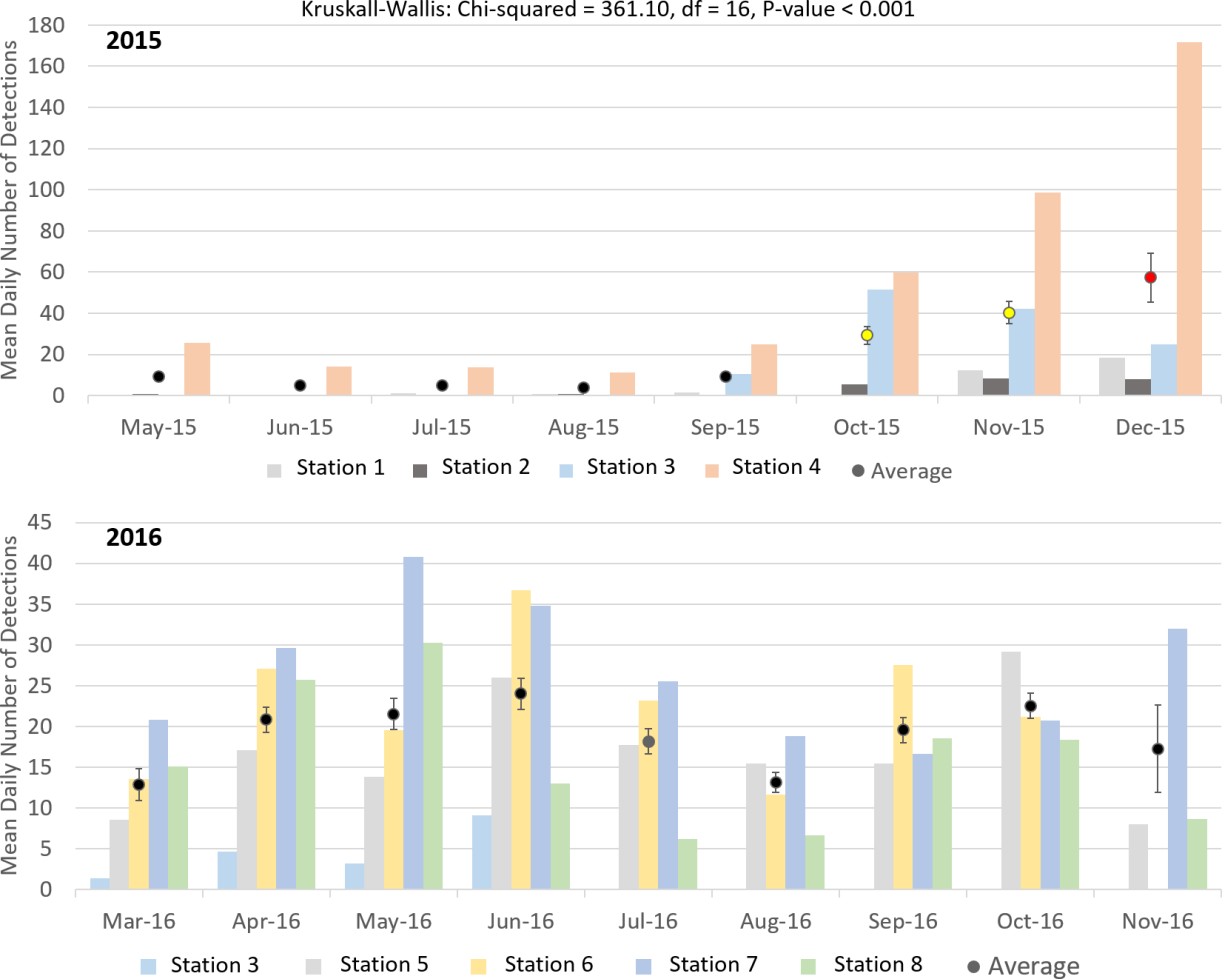
Mean daily number of Cuvier’s beaked whale detections per month for each station that recorded data in 2015 (top) and 2016 (bottom), as well as the average across all stations with standard error bars. Results of Kruskall-Wallis tests are given and results from Tukey-Kramer multiple comparisons tests (applied to averages from 2015 and 2016 separately) are represented by coloured means with different colours denoting statistically significant differences.

Cuvier’s beaked whale clicks occurred throughout the recording period at all stations with the species present on 72.8% of recording days (Fig 3 and Table 4). MANOVA results indicate that the mean daily number of Cuvier’s beaked whale detections was significantly different between monitoring stations and between months of the year (Table 5). Indeed, station 4, at the northern edge of Porcupine Bank, had the highest mean daily Cuvier’s detections while the lowest mean detections per day were observed at the northernmost stations 1 and 2 (Fig 4). Intermediate detection rates occurred at stations 3 and 5 to 8 (Fig 4). In 2015, when stations were in the northern half of the study area, the mean daily detections were higher on average in the months of October, November, and December than from May to September (Fig 5); a pattern driven by the high number of detections at station 4 in the winter of 2015. December 2015 had more detections than any other month of that year (Fig 5). In contrast, in 2016 when stations were deployed further south, Cuvier’s beaked whale mean daily detections increased through the spring, decreased in the summer, and increased again in the autumn (Fig 5). However, these trends were not statistically significant (Fig 5).

**Table 4:** Percentage of days with Cuvier’s beaked, Sowerby’s beaked, and northern bottlenose whale clicks recorded at 8 stations off western Ireland in 2015 and 2016.

Station	Cuvier’s beaked whale		Sowerby’s beaked whale	Northern bottlenose whale
**1**	30.4		84.1	0.5
**2**	20.6		57.0	0.0
**3**	63.9		75.6	0.5
**4**	87.3		75.1	2.8
**5**	93.8		69.2	0.9
**6**	97.1		53.8	0.5
**7**	98.3		38.3	0.0
**8**	87.0		18.7	0.0

**Table 5:** Results of Cuvier’s MANOVA.

Variable(s)	Sum of Squares	df	F-value	P-value
Month	2.43x10^5^	16	25.5	<0.001
Station	3.06x10^5^	7	73.3	<0.001
Seismic	118	1	0.20	0.66
Month, Station	2.68x10^5^	44	10.2	<0.001
Month, Seismic	3.46x10^3^	12	0.48	0.93
Station, Seismic	1.33 x10^3^	4	0.56	0.69
Month, Station, Seismic	909	4	0.38	0.82

Includes the sum of squares, degrees of freedom (df), F-value, and P-value where the response variable was the mean number of Cuvier’s beaked whale detections per day and the statistical significance level was <0.05.

**Fig 6:**
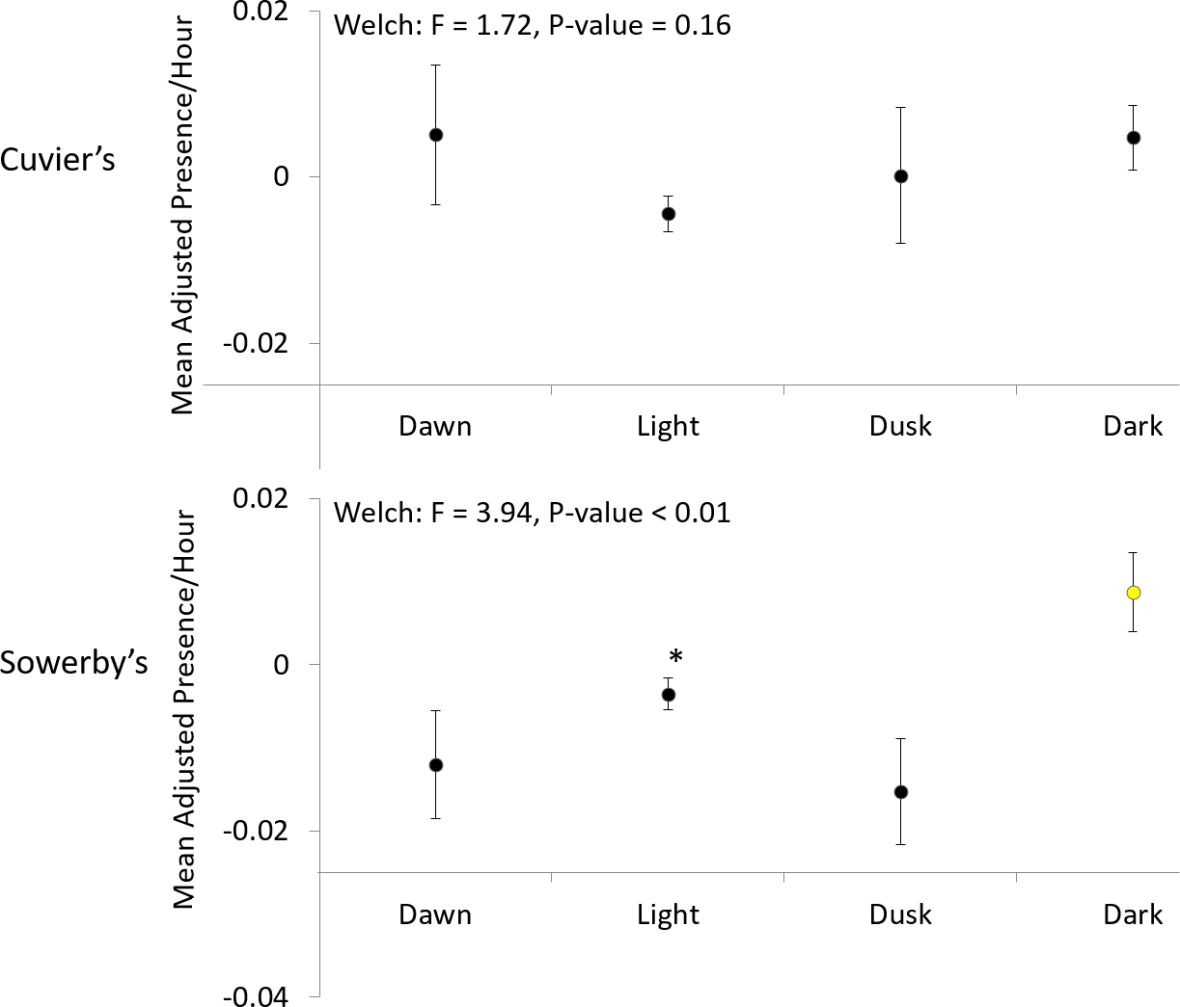
Mean adjusted hours with Cuvier’s (top) and Sowerby’s (bottom) beaked whale clicks with standard error bars in four light periods: dawn, light, dusk, and dark. Light period times were obtained from *Reda and Andreas (62)*. Results of Welch’s ANOVA are given for each species and results from Tukey-Kramer multiple comparisons tests are represented by coloured means with different colours denoting statistically significant differences. *The Sowerby’s light period does not differ significantly from any of the other light periods.

The adjusted mean hours with Cuvier’s beaked whale clicks were highest during dark (4.7x10^-3^; SE = 3.9x10^-3^) and lowest during light (-4.4x10^-3^; SE = 4.1x10^-3^) with dawn and dusk values intermediate at 0.005 (SE = 8.4x10^-3^) and 1.8x10^-4^ (SE = 8.1x10^-3^), respectively (Fig 6). All light periods had a sample size (n) of 1270. The null hypothesis that the medians of the mean hours with Cuvier’s beaked whale clicks did not differ between the four light periods was not rejected by Welch’s ANOVA (Fig 6).

### Sowerby’s beaked whales

**Fig 7:**
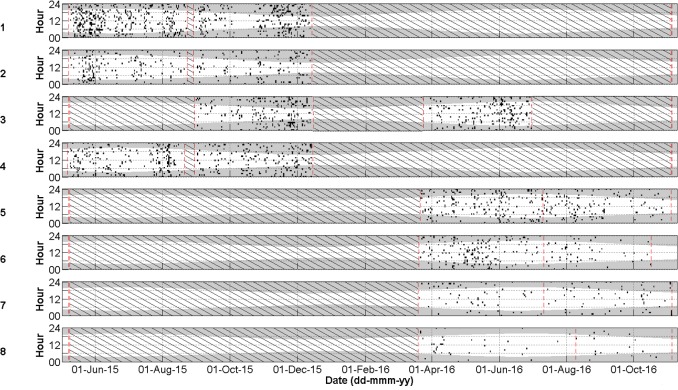
Daily and hourly occurrence of Sowerby’s beaked whale clicks per acoustic file at stations 1–8 represented as individual black dots (P = 0.96, R = 0.93). Shaded areas indicate periods of darkness. The red dashed lines indicate the deployment and retrieval dates. Black diagonal lines indicate periods with no data.

**Fig 8:**
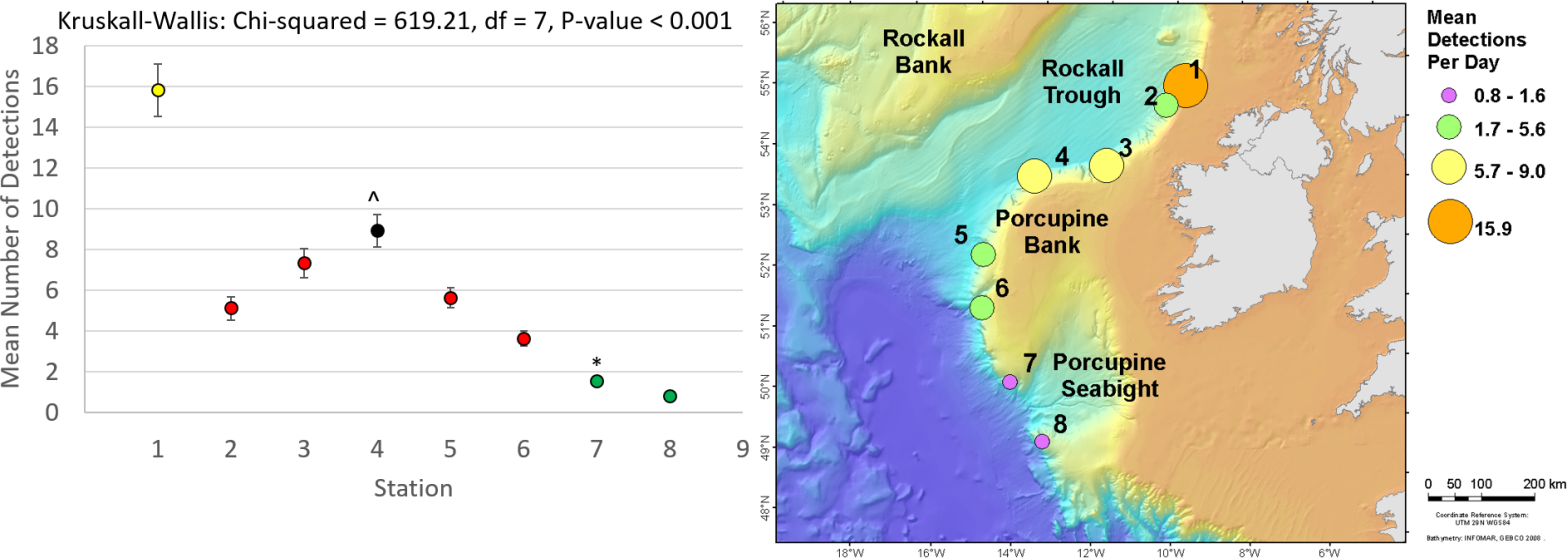
**Mean Sowerby’s beaked whale detections per recording day bubble plot (right) and with standard error bars (left) for stations 1 (SE = ±1.28), 2 (SE = ±0.56), 3 (SE = ±0.70), 4 (SE = ±0.81), 5 (SE = ±0.51), 6 (SE = ±0.37), 7 (SE = ±0.23), and 8 (SE = ±0.15) off western Ireland from May to Dec 2015 (stations 1–4) and Mar to Nov 2016 (stations 3 and 5–8).** Results of Kruskall-Wallis tests are given and results from Tukey-Kramer multiple comparisons tests are represented by coloured means (left) with different colours denoting statistically significant differences. ^Station 4 does not differ from 3 or 5. *Station 7 does not differ from 8 or 6, but 8 differs from 6.

**Fig 9:**
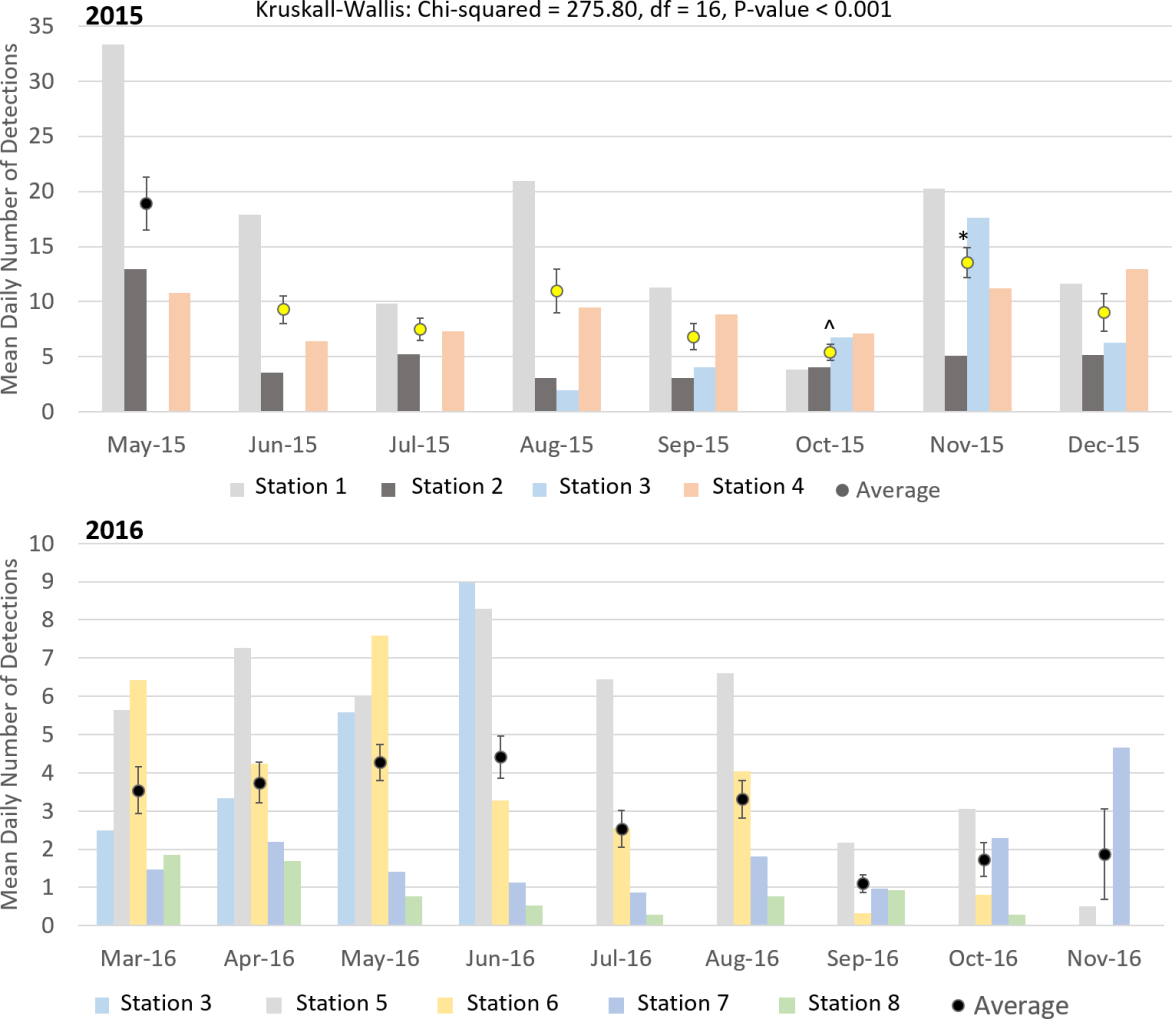
Mean daily number of Sowerby’s beaked whale detections per month for each station that recorded data in 2015 (top) and 2016 (bottom) as well as the average across all stations with standard error bars. Results of Kruskall-Wallis tests are given and results from Tukey-Kramer multiple comparisons tests (applied to average of 2015 and 2016 separately) are represented by coloured means with different colours denoting statistically significant differences. *November 2015 differs from July and October 2015, but not from June, August, September, or December 2015. ^October 2015 differs from August 2015.

Sowerby’s beaked whale clicks were present at all stations in 2015 and 2016, occurring on 58.4% of recording days (Fig 7 and Table 4). Like Cuvier’s beaked whales, Sowerby’s beaked whale clicks showed spatio-temporal variation with the mean number of detections per day being significantly different between months of the year and between stations (Table 6). The northernmost station 1 had significantly higher mean daily Sowerby’s beaked whale detections than all other stations while station 8 had fewer detections than all stations but 7 (Fig 8). Station 4 has more detections than stations 2, 6, 7 and 8 (Fig 8). In 2015 when the four northern stations were monitored, the mean daily detections were highest on average in May, a pattern largely driven by the high number of detections at station 1 (Fig 9). November had significantly more detections than July, September, and October, with the detection rate in October also being significantly lower than August (Fig 9). On average in 2016 when the southern stations were monitored, there was no significant difference in the number of Sowerby’s beaked whale detections between months (Fig 9).

**Table 6:** Results of Sowerby’s MANOVA.

Variable(s)	Sum of Squares	df	F-value	P-value
Month	1.36x10^4^	16	10.4	<0.001
Station	1.60x10^4^	7	27.8	<0.001
Seismic	152	1	1.85	0.17
Month, Station	1.01x10^4^	44	2.79	<0.001
Month, Seismic	878	10	1.07	0.38
Station, Seismic	1.04 x10^3^	6	2.10	0.05
Month, Station, Seismic	960	17	0.69	0.82

Includes the sum of squares, degrees of freedom (df), F-value, and P-value where the response variable was the mean number of Sowerby’s beaked whale detections per day and the statistical significance level was <0.05.

The mean adjusted average hours with Sowerby’s beaked whale clicks were highest during dark (8.8x10^-3^; SE = 4.7x10^-3^) and lowest during dawn (-0.01; SE = 6.5x10^-3^) and dusk (-0.02; SE = 6.4x10^-3^) with light intermediate at 3.5x10^-3^ (SE = 1.9x10^-3^; Fig 6). All light periods had a sample size (n) of 1019. The null hypothesis that the medians of the mean adjusted hours with Sowerby’s beaked whale clicks did not differ between the four light periods was rejected (Fig 6). The mean adjusted hours with Sowerby’s clicks during dark was significantly higher than during dawn and dusk, but no different than light. No significant difference in the means was observed between dawn, dusk, or light (Fig 6).

### Northern bottlenose whales

**Fig 10:**
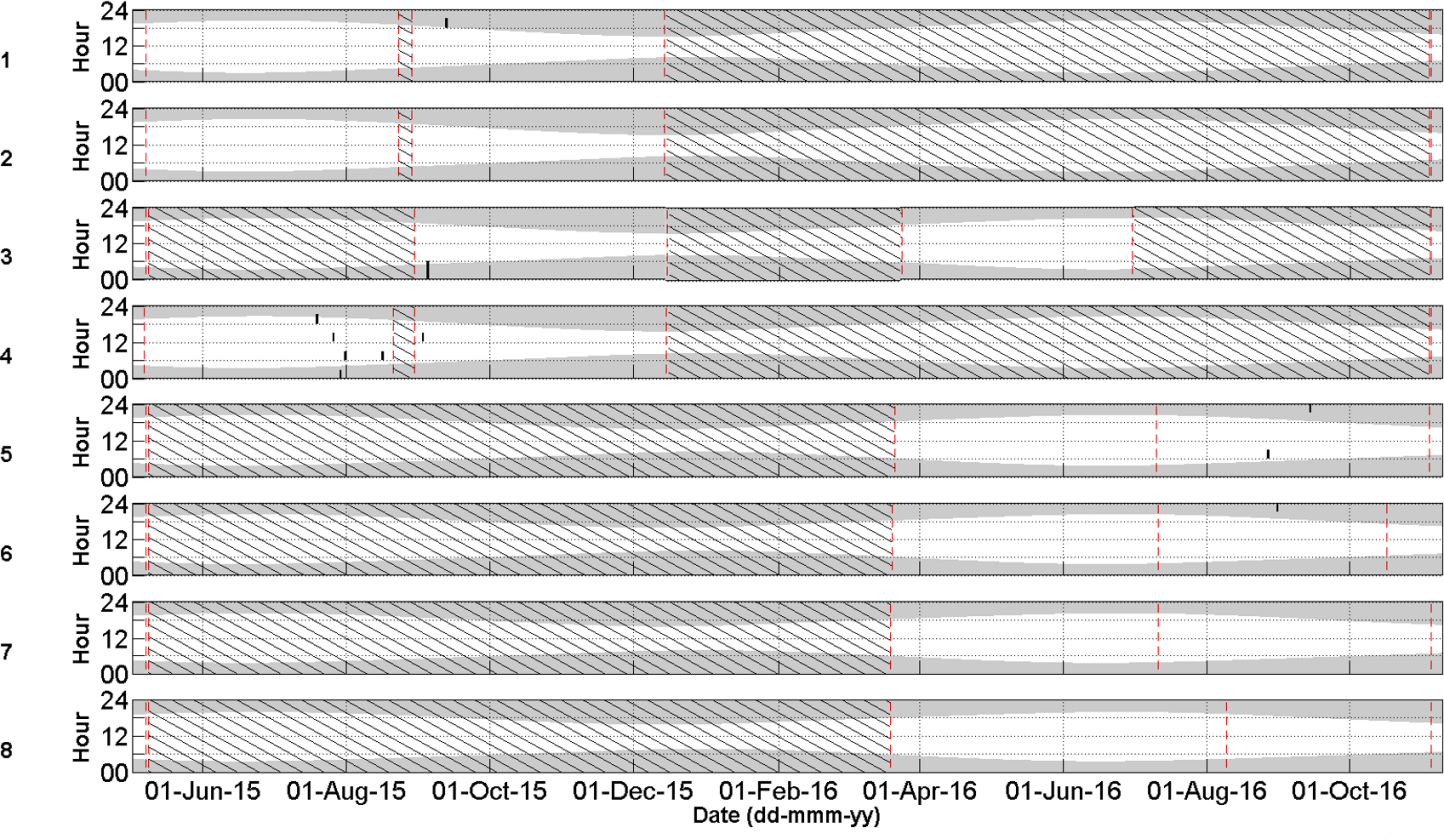
Daily and hourly occurrence of northern bottlenose whale clicks per acoustic file at stations 1–8 represented as individual black dots (P = 1, R = 0.42). Shaded areas indicate periods of darkness. The red dashed lines indicate the deployment and retrieval dates. Black diagonal lines indicate periods with no data.

Northern bottlenose whale clicks were rare, only occurring in the summer and autumn on 1.0% of recording days (Fig 10 and Table 4). In 2015 clicks were recorded on 19, 26, 29, 31 Jul, 16 Aug, and 2 Sept at station 4, 4 Sept at station 3 and on 12 Sept at station 1. In 2016 northern bottlenose whale clicks occurred at station 5 on 26 Aug and 13 Sept and at station 6 on 30 Aug. They were never automatically or manually detected at stations 2, 7, or 8 and were absent in the acoustic dataset from the months of April to June and from October to December.

### Seismic impulses

**Fig 11:**
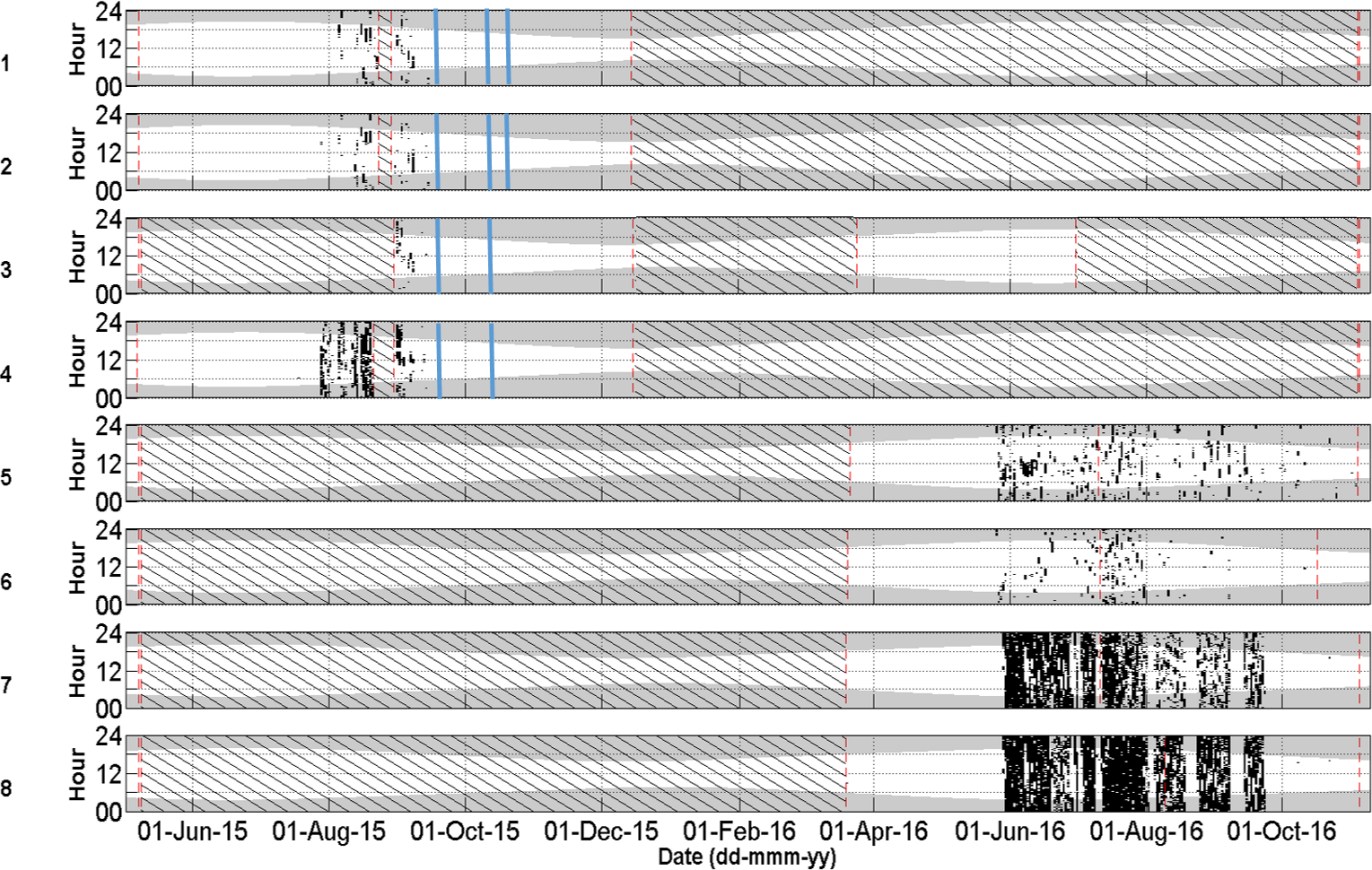
Daily and hourly occurrence of seismic impulses (black dots) at stations 1–8. Shaded areas indicate periods of darkness. The red dashed lines indicate the deployment and retrieval dates. Black diagonal lines indicate periods with no data. Blue solid lines indicate days when sonar was opportunistically observed.

Seismic impulse detections occurred at all stations in 2015 and 2016 almost exclusively from June to October, but were most prominent at the more southerly stations 7 and 8 (Fig 11). Indeed, from 1 June to 1 October, seismic impulse detections occurred on 49.4% of recording days (Fig 11; 19.1% at station 1, 20.0% at station 2, 16.7% at station 3, 30.4% at station 4, 76.2% at station 5, 45.9% at station 6, 82.8% at station 7, and 82.8% at station 8). Seismic survey pulses occurred in bouts with gaps of up to a few days duration. MANOVA results indicate that the mean number of acoustic files with Cuvier’s beaked whale clicks were not significantly different between days when seismic was present against days when seismic was absent (Table 5). Nor was there a significant difference between the mean number of acoustic files with Sowerby’s clicks when seismic was present versus absent (Table 6).

**Fig 12:**
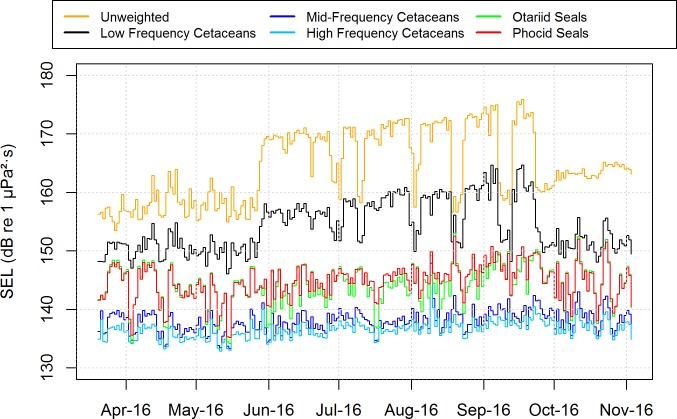
Daily unweighted and marine-mammal auditory filter weighted *[66]* sound exposure levels for station 8 which had the highest seismic sound levels across all eight deployments.

Station 8 had the highest level of seismic activity (Fig 11). The daily sound exposure levels were calculated for station 8, both unweighted and using the marine-mammal auditory weighting functions suggested in [65] (Fig 12). The unweighted sound exposures increased from the range of 154–163 dB re 1 μPa^2^·s before seismic started to 165–175 dB re 1 μPa^2^·s when seismic was present. Most of the seismic energy was in the lowest frequency bands and did not increase the daily sound exposure level when the sound was weighted for the mid and high frequency cetacean groups, which includes the beaked whales. The sound exposure levels never approached the thresholds for possible temporary auditory injury to any of the marine mammal groups [66].

## Discussion

Three beaked whale species were acoustically present in the northern Atlantic Margin off western Ireland in 2015 and in the southern Atlantic Margin in 2016. During both monitoring periods, Cuvier’s and Sowerby’s beaked whale clicks were common while northern bottlenose whale clicks were rare. Although the interpretation of spatial and temporal acoustic occurrence patterns presented here is somewhat obscured by the fact that the whole area was not sampled within the same calendar year and winter months were not sampled beyond early to mid-December 2015 and early November 2016, this study provides valuable new data in space and time regarding beaked whale occurrence in this part of the North-East Atlantic. It also provides the first insights into the importance of the Atlantic Margin for Cuvier’s and Sowerby’s beaked whales and establishes a baseline for beaked whale monitoring and assessment off western Ireland.

The static acoustic recorders had a limited beaked whale detection range since Ziphiid acoustic signals are high frequency in nature and thus attenuate quickly with distance in the water column, even in ideal acoustic conditions. For example, it is predicted that most Ziphiids cannot be detected beyond 4 km [67], although Blainville’s beaked whales have been detected up to 6.5 km in the Bahamas [68]. Sound propagation modeling within the Porcupine Seabight off southwest Ireland predicted that Cuvier’s and Sowerby’s beaked whales cannot be detected beyond 14 km and 4 km, respectively (Sam Denes, personal communication). Northern bottlenose whales have an estimated detectability radius of up to 8 km in a submarine canyon off eastern Canada (Sam Denes, personal communication). Therefore, the beaked whales detected in the present study were certainly within 14 km of the recording stations, and more likely within 4 to 5 km most of the time. We can be confident that acoustic signals recorded at a station were not simultaneously recorded by others since the nearest stations (stations 1 and 2) were situated over 50 km apart. Due to the high beaked whale click frequencies, no seasonal differences in detection range due, to increased background noise in winter, for example, are expected [69].

Cuvier’s beaked whales are one of the most widespread and abundant beaked whale species globally. In Ireland, they have the largest latitudinal range and are the most frequently stranded Ziphiid [3, 9, 70]. Although their presence in the Atlantic Margin region was expected, the breadth and regularity of their occurrence were novel and somewhat unexpected given the limited sightings and acoustic detections during previous vessel-based visual and towed array surveys [3, 33, 38, 71]. Indeed, in a previous offshore study, O’Cadhla et al. [33] described only three sightings of this species: two in the Rockall Trough in August 2000 and one along the southern slope of the Porcupine Bank in August 2001. In a parallel towed acoustic study, Aguilar de Soto et al. [38] reported only a few Cuvier’s beaked whale detections along the Atlantic Margin. Furthermore, an extensive northeast Atlantic visual survey found Cuvier’s beaked whales to occur almost exclusively in the Bay of Biscay [4] to the south of our study area. The conflicting results between study methods highlight the necessity of utilizing PAM when studying such elusive animals.

Keeping in mind the caveats caused by the asynchronous sampling of northern and southern areas, Cuvier’s beaked whales seemed to show a preference for more southern stations along the continental shelf edge from the northern slope of the Porcupine Bank to the southern entrance of the Porcupine Seabight, a pattern likely linked to their prey distribution. In European waters, the diet of Cuvier’s beaked whale is dominated by cephalopods including *Teuthowenia megalops*, *Taonius pavo*, *Histioteuthis reversa*, *Mastigoteuthis*, *Gonatus*, and *Chiroteuthis* species [72–74]. *Teuthowenia megalops* is abundant and widely distributed in deep waters of the North Atlantic [75] and was the most abundant species of Cranchiid squid caught off Ireland by Collins et al. [76]. *Histioteuthis reversa* is a pelagic species found in the North Atlantic from 11° to 59°N and has been recorded in water depths of 1343–1794 m [77, 78]. These squid are thought to occur in higher abundance over deep slope areas and have been found to spawn over the continental slope in the North Atlantic [75]. In the eastern North Atlantic, *Taonius pavo* has been found from 28° to 47° N [78]. Collectively, these cephalopods show a latitudinal trend in diversity whereby the number of species approximately doubles from higher latitudes approximately 60°N to lower latitudes around 30°N [78, 79]. Cuvier’s beaked whale movements may be influenced by this latitudinal increase in prey diversity.

The lack of a conclusive diel pattern in Cuvier’s beaked whale clicks suggests that, in this continental slope region, they forage at similar rates throughout the day and night as the majority of Cuvier’s beaked whale acoustic activity has been linked to foraging dives [54]. Previous work on diel acoustic behaviour of beaked whales has revealed a range of results. Unknown beaked whales off the Hawaiian islands of Kauai and Ni’ihau and beaked whales, including Cuvier’s, recorded in the Cross Seamount of Hawaii clicked predominantly at night [15, 80, 81]. A more common finding is that beaked whales have different diel clicking behaviour depending on location and time of year [12, 14, 15]. For example, in the Ligurian Sea, Giorli et al. [14] described Cuvier’s beaked whales foraging more at night through July, early August, and September, but in late August, October, and November there was no diel pattern. Furthermore, Giorli et al. [12] monitored three sites around the island of Hawaii and found that beaked whales clicked more at night at one site, more during the day at another, and had no diel pattern at the third site. While beyond the scope of our current work, further investigation into the present data at a station-specific and time-specific level could be revealing.

Cuvier’s beaked whale clicks were particularly common off the northwestern slope of Porcupine Bank, a finding counter to a potential southern bias in distribution. Previous work in this Atlantic Margin region highlighted the potential importance for cetacean diversity of the northern and western margins of the Porcupine Bank [33]. The oceanography of this continental slope area leading into the eastern Rockall Trough may be uniquely productive and habitat modelling in the future could be revealing.

Strandings of Cuvier’s beaked whales in Ireland are highest in spring and early summer and lowest in winter [82]. This led to an earlier hypothesis that Cuvier’s beaked whales move northward into Irish waters between January and March to the northern limits of their range in spring and summer [82]. Our data do not support such a migration as Cuvier’s beaked whale occurrence was constant regardless of time of year except for October to December in 2015 when the species’ occurrence increased as winter approached, particularly off the northwest slope of the Porcupine Bank. The results suggest that through late autumn 2015, individuals from outside of our monitoring areas moved into the northwest Porcupine Bank slope region, possibly driven by increased prey, or less likely, that the animals already in the area began producing more clicks. PAM data through the entire winter is required to further explore Cuvier’s beaked whale temporal occurrence in the Atlantic Margin.

Prior to this study, Sowerby’s beaked whale occurrence in Irish waters was largely unknown and had been limited to a small number of visual sightings [33, 47, 83, 84]. We confirm Sowerby’s beaked whale presence and provide the first description of their occurrence across the slopes of the Atlantic Margin throughout the monitored periods. The preference of Sowerby’s beaked whales for northern stations observed here is in agreeance with findings from an extensive North-East Atlantic visual survey which suggested that the species is more prominent north of 57°N [4]. Indeed, it seems that Sowerby’s beaked whale densities are higher north of 50^O^ N in the eastern North Atlantic, although sporadic sightings in the Bay of Biscay [4] and in the Canary Islands [85] indicate that the species’ overall range extends further south. Sowerby’s beaked whale spatial occurrence deviated from the generalised northern distribution at the northwest margin of the Porcupine Bank where, like Cuvier’s beaked whales, occurrence was notably high.

The distribution of Sowerby’s beaked whales is likely linked to prey that are typically smaller than those of Cuvier’s beaked and Northern bottlenose whales [86]. In European waters, fish are their major prey item with Gadidae and Merlucciidae being the most important [73, 87, 88], though *Micromesistius poutassou*, *Trisopterus luscus/minutus* and *Merluccius merluccius* have also been identified as significant prey in the Bay of Biscay [87]. The increased Sowerby’s beaked whale clicks during hours of darkness is likely driven by prey availability since daily variations in the vertical distribution of offshore pelagic fish species is well documented [89]. For example, *Merluccius merluccius*, *Micromesistius poutassou and Trisopterus* species undergo diel vertical migration and travel from the seabed to the surface at night [89–91]. As mentioned, beaked whale nighttime foraging has been reported previously [15, 80, 81].

Sowerby’s beaked whale occurrence was constant through much of the 2015 and 2016 monitoring periods, apart from a large peak in May 2015 at the northernmost stations and a subtler peak in Nov 2015 along the northern Porcupine Bank slope. These findings support previous suggestions that at least some Sowerby’s beaked whales move northwards in late winter and spring and southwards in the autumn [82], though our lack of data over the entire study area during the same time-frame limits our ability to make conclusive migratory conclusions.

The infrequent occurrence of northern bottlenose whales in the present study reflects previous findings as fewer than ten sightings of this species have been recorded along Ireland’s Atlantic Margin [3, 4, 47, 92]. Northern bottlenose whale occurrence in Ireland is mostly known from occasional stranding events, the majority of which have occurred between the months of July and October, a similar time-frame as the clicks detected in our data [82]. In the North-East Atlantic, Northern bottlenose whales are thought to undertake annual migrations. They move to Arctic waters in spring to forage on newly hatched cephalopods (*Gonatus fabricii)* that do not occur south of 55°N [72, 75] and, in early autumn, they return to temperate waters to forage on Cranchiidae such as *T*. *pavo* and *Teuthowenia megalops* [86, 93, 94]. The continental slope habitats monitored in this study do not appear to be part of the core habitat of the species in this part of the North-East Atlantic. One plausible scenario is that the Atlantic Margin lies at the boundary of the species’ current distributional range and is therefore only occasionally frequented by migrating or foraging individuals. The majority of Northern bottlenose whales may occur further offshore in the deeper waters of the Rockall Trough where the species has been more regularly sighted [4] and detected acoustically [95]. The steep eastern slope of the Rockall Bank may also offer a suitable habitat for this species [96, 97].

The absence of Gervais’ and True’s beaked whale clicks in our data indicates that these species were either truly absent from the region or that they occur in the region but were not identified in the acoustic analyses. Both species are known in Irish waters from very rare instances [33, 40]; thus, the former may be the case, but we cannot rule out the latter. Indeed, the acoustic signals of Gervais’ beaked whales are poorly understood and True’s beaked whale clicks have yet to be described. Our automated detector was not trained to detect these species and given that manual analysis was driven by automated detections, acoustic signals of these species may well have occurred in files that went unanalyzed. As new research on the repertoire of beaked whales continues to emerge, this dataset can be revisited in the future to more confidently determine the presence or absence of Gervais’ and True’s beaked whales in the Atlantic Margin.

Though not the aim of the present study, it is nevertheless pertinent to describe the anthropogenic sounds we encountered during analysis given the risks that some human activities can introduce to beaked whales [17–26]. The seismic impulses recorded in 2015 were produced by an unknown seismic exploration program, likely distant and offshore. In 2016, seismic impulses were produced by a licensed seismic exploration program for hydrocarbons in the Porcupine Seabight that occurred 165 to 230 km from stations 3 and 5 to 8. Seismic occurrence was most frequent at stations 7 and 8, at the mouth of the Porcupine Seabight, (Fig 11) because the other stations were shadowed by the shelf. The absence of a discernable Cuvier’s and Sowerby’s beaked whale acoustic behavioural reaction in terms of click presence/absence and the low level of received seismic energy suggests little impact from seismic activity at these received levels [98]. The detection of naval sonar pulses was of concern, due to its association with previous stranding events by deep-diving species. The use of naval sonar was opportunistically observed during three periods in the autumn of 2015: 17–18 Sept at Stations 1–4, 7–10 Oct at Stations 1–4, and 19–20 Oct at Stations 1 and 2 (Fig 11). Detailed acoustic behavioural response analysis for seismic airguns and the sonar pulses was beyond the scope of our work, but should be explored in the future.

While PAM on the Atlantic Margin has allowed us to document the occurrence of elusive beaked whales at relatively low effort and cost in a remote offshore region, PAM has limitations. The bottom-mounted acoustic recorders are likely only detecting clicks during deep foraging dives. Thus, missing animals in the area, but at the surface, those who dive but do not actively echolocate, or those who click at an angle or distance from the recorder such that the received clicks cannot be identified by either the automated detector or analysts. Therefore, the results presented here represent a minimum occurrence of these species in offshore Irish waters. Given the broad spatio-temporal scale of our data, the potential influence of fluctuations in dive rate, click rate, and animal movements in or out of the detection area should be negligible, as the resulting upward or downward biases on detection rates should even out over time. This assumes a similar use of the habitat surrounding the recording locations, since differences in dive and click rate as well as residency patterns may be associated with differences in habitat use [12]. Therefore, despite the accepted limitations of PAM for odontocete clicks, we believe that these results depict an accurate picture of the relative occurrence of beaked whales across the monitored stations and can be used as a baseline for future assessments of occurrence and habitat use.

## Conclusions

The findings presented here highlight the hitherto unknown importance of the Atlantic Margin for Cuvier’s and Sowerby’s beaked whales and provide the first insights into potential range differences, with each species respectively favouring slope areas in the southern and northern parts of the study area, a pattern likely driven by preferred prey distributions. In contrast, northern bottlenose whales only occurred sporadically in the late summer and early autumn. All observed species showed preference for the northwest margin of Porcupine Bank, highlighting a potentially important biological region in need of further research. Our acoustically-derived results are in stark contrast to the scarcity of sightings of beaked whales off Ireland from traditional ship- or boat-based survey methods, which strongly supports the use of static acoustic monitoring as a reliable method for detecting these elusive species. Given the known sensitivity of these animals to some anthropogenic sounds, our findings should be given appropriate consideration when assessing the environmental risks and management actions required for any expansion of human activities in Ireland’s Atlantic Margin.

## Supporting information

S1 Dataset Period1, station1 automated detections for Cuvier’s beaked and northern bottlenose whales. (CSV)

S2 Dataset Period 1, station1 automated detections for Sowerby’s beaked whales. (CSV)

S3 Dataset Period 2, station2 automated detections for Cuvier’s beaked and northern bottlenose whales. (CSV)

S4 Dataset Period 2, station3 automated detections for Cuvier’s beaked and northern bottlenose whales. (CSV)

S5 Dataset Period 2, station4 automated detections for Cuvier’s beaked and northern bottlenose whales. (CSV)

S6 Dataset Period 1, station2 automated detections for Cuvier’s beaked and northern bottlenose whales. (CSV)

S7 Dataset Period 1, station2 automated detections for Sowerby’s beaked whales. (CSV)

S8 Dataset Period 1, station4 automated detections for Cuvier’s beaked and northern bottlenose whales. (CSV)

S9 Dataset Period 1, station4 automated detections for Sowerby’s beaked whales. (CSV)

S10 Dataset Period 2, station1 automated detections for Cuvier’s beaked and northern bottlenose whales. (CSV)

S11 Dataset Period 2, station1 automated detections for Sowerby’s beaked whales. (CSV)

S12 Dataset Period 2, station2 automated detections for Sowerby’s beaked whales. (CSV)

S13 Dataset Period 2, station3 automated detections for Sowerby’s beaked whales. (CSV)

S14 Dataset Period 2, station4 automated detections for Sowerby’s beaked whales. (CSV)

S15 Dataset Period 3, station3 automated detections for Cuvier’s beaked and northern bottlenose whales. (CSV)

S16 Dataset Period 3, station5 automated detections for Cuvier’s beaked and northern bottlenose whales. (CSV)

S17 Dataset Period 3, station6 automated detections for Cuvier’s beaked and northern bottlenose whales. (CSV)

S18 Dataset Period 3, station7 automated detections for Cuvier’s beaked and northern bottlenose whales. (CSV)

S19 Dataset Period 3, station8 automated detections for Cuvier’s beaked and northern bottlenose whales. (CSV)

S20 Dataset Period 3, station3 automated detections for Sowerby’s beaked whales. (CSV)

S21 Dataset Period 3, station5 automated detections for Sowerby’s beaked whales. (CSV)

S22 Dataset Period 4, station7 automated detections for Cuvier’s beaked and northern bottlenose whales. (CSV)

S23 Dataset Period 4, station8 automated detections for Cuvier’s beaked and northern bottlenose whales. (CSV)

S24 Dataset Period 4, station5 automated detections for Sowerby’s beaked whales. (CSV)

S25 Dataset Period 4, station6 automated detections for Sowerby’s beaked whales. (CSV)

S26 Dataset Period 4, station7 automated detections for Sowerby’s beaked whales. (CSV)

S27 Dataset Period 4, station8 automated detections for Sowerby’s beaked whales. (CSV)

S28 Dataset Period 3, station6 automated detections for Sowerby’s beaked whales. (CSV)

S29 Dataset Period 3, station7 automated detections for Sowerby’s beaked whales. (CSV)

S30 Dataset Period 3, station8 automated detections for Sowerby’s beaked whales. (CSV)

S31 Dataset Period 4, station5 automated detections for Cuvier’s beaked and northern bottlenose whales. (CSV)

S32 Dataset Period 4, station6 automated detections for Cuvier’s beaked and northern bottlenose whales. (CSV)

S33 Dataset Manual verification of automated detections for Cuvier’s beaked and northern bottlenose whales. (CSV)

S34 Dataset Manual verification of automated detections for Sowerby’s beaked whales. (CSV)

## References

[1] Hooker SK, Whitehead H, Gowans S. Ecosystem consideration in conservation planning: energy demand of foraging bottlenose whales (*Hyperoodon ampullatus*) in a marine protected area. Biol Conserv. 2002;104(1):51–8.

[2] Stanistreet JE, Nowacek DP, Baumann-Pickering S, Bell JT, Cholewiak DM, Hildebrand JA, et al Using passive acoustic monitoring to document the distribution of beaked whale species in the western North Atlantic Ocean. Can J Fish Aquat Sci. 2017;(999):1–12.

[3] Berrow S, Whooley P, O’Connell M, Wall D. Irish Cetacean Review (2000–2009). Irish Whale and Dolphin Group, 60pp. 2010.

[4] Rogan E, Cañadas A, Macleod K, Santos MB, Mikkelsen B, Uriarte A, et al Distribution, abundance and habitat use of deep diving cetaceans in the North-East Atlantic. DSR. 2017.

[5] Moore JE, Barlow JP. Declining abundance of beaked whales (Family Ziphiidae) in the California current large marine ecosystem. PLoS ONE. 2013;8(1):e52770 doi: 10.1371/journal.pone.0052770 23341907 10.1371/journal.pone.0052770 PMC3547055

[6] Wall Ded, O'Brien J, Meade J, Allen BM, editors. Summer distribution and relative abundance of cetaceans off the west coast of Ireland. Biology and Environment: Proceedings of the Royal Irish Academy; 2006: JSTOR.

[7] MacLeod CD, Mitchell G. Key areas for beaked whales worldwide. J Cetacean Res Manage. 2006;7(3):309–22.

[8] Jefferson TA, Webber MA, Pitman RL. Marine Mammals of the World, A Comprehensive Guide to their Identification. Amsterdam: Elsevier; 2008.

[9] Macleod CD. Review of the distribution of Mesoplodon species (order Cetacea, family Ziphiidae) in the North Atlantic. Mamm Rev. 2000;30(1):1–8.

[10] Cañadas A, Sagarminaga R, Garcıa-Tiscar S. Cetacean distribution related with depth and slope in the Mediterranean waters off southern Spain. Deep Sea Research Part I: Oceanographic Research Papers. 2002;49(11):2053–73.

[11] Moulins A, Rosso M, Nani B, Würtz M. Aspects of the distribution of Cuvier's beaked whale (*Ziphius cavirostris*) in relation to topographic features in the Pelagos Sanctuary (north-western Mediterranean Sea). J Mar Biol Assoc UK. 2007;87(1):177–86.

[12] Giorli G, Neuheimer A, Copeland A, Au WW. Temporal and spatial variation of beaked and sperm whales foraging activity in Hawai'i, as determined with passive acoustics. The Journal of the Acoustical Society of America. 2016;140(4):2333–43. doi: 10.1121/1.4964105 27794335 10.1121/1.4964105

[13] Giorli G, Au WW, Ou H, Jarvis S, Morrissey R, Moretti D. Acoustic detection of biosonar activity of deep diving odontocetes at Josephine Seamount High Seas Marine Protected Area. The Journal of the Acoustical Society of America. 2015;137(5):2495–501. doi: 10.1121/1.4919291 25994682 10.1121/1.4919291

[14] Giorli G, Au WW, Neuheimer A. Differences in foraging activity of deep sea diving odontocetes in the Ligurian Sea as determined by passive acoustic recorders. Deep Sea Research Part I: Oceanographic Research Papers. 2016;107:1–8.

[15] Baumann-Pickering S, Roch MA, Brownell RL Jr, Simonis AE, McDonald MA, Solsona-Berga A, et al Spatio-temporal patterns of beaked whale echolocation signals in the North Pacific. PLoS ONE. 2014;9(1):e86072 doi: 10.1371/journal.pone.0086072 24465877 10.1371/journal.pone.0086072 PMC3899217

[16] [IUCN] International Union on the Conservation of Nature. The IUCN Red List of Threatened Species: beaked whales 2017 [cited 2017 24 November]. Available from: http://www.iucnredlist.org/search.

[17] Cox TM, Ragen T, Read A, Vos E, Baird R, Balcomb K, et al Understanding the impacts of anthropogenic sound on beaked whales. J Cetacean Res Manage. 2006;7(3):177–87.

[18] Frantzis A. Does acoustic testing strand whales? Nature. 1998;392(6671):29–. doi: 10.1038/32068 9510243 10.1038/32068

[19] Fernández A, Edwards JF, Rodriguez F, De Los Monteros AE, Herraez P, Castro P, et al Gas and fat embolic syndrome involving a mass stranding of beaked whales (Family Ziphiidae) exposed to anthropogenic sonar signals. Vet Pathol. 2005;42(4):446–57. PubMed PMID: ISI:000230282600006. doi: 10.1354/vp.42-4-446 16006604 10.1354/vp.42-4-446

[20] England GR, Evans D, Lautenbacher C, Morrissey S, Hogarth W. Joint Interim Report Bahamas Marine Mammal Stranding Event of 15–16 March 2000. US Department of Commerce, US Secretary of the Navy 2001.

[21] Evans PG, Miller LA, editors. Active sonar and cetaceans. Proceedings of workshop held at the ECS 17th annual conference, Las Palmas, 8th March; 2003.

[22] Fernández A. Beaked whale (Ziphius cavirostris) mass stranding on Almería’s coasts in southern Spain, 26–27 January 2006. Report of the University of Las Palmas de Gran Canaria, Canary Islands. 2006;24.

[23] Arbelo M, De Quiros YB, Sierra E, Méndez M, Godinho A, Ramírez G, et al Atypical beaked whale mass stranding in Almeria's coasts: pathological study. Bioacoustics. 2008;17(1–3):294–7.

[24] Falcone EA, Schorr GS, Watwood SL, DeRuiter SL, Zerbini AN, Andrews RD, et al Diving behaviour of Cuvier's beaked whales exposed to two types of military sonar. Royal Society Open Science. 2017;4(8):170629 doi: 10.1098/rsos.170629 28879004 10.1098/rsos.170629 PMC5579120

[25] Filadelfo R, Mintz J, Michlovich E, D'Amico A, Tyack PL, Ketten DR. Correlating military sonar use with beaked whale mass strandings: what do the historical data show? Aquat Mamm. 2009;35(4):435.

[26] D'Amico A, Gisiner RC, Ketten DR, Hammock JA, Johnson C, Tyack PL, et al Beaked whale strandings and naval exercises. Aquat Mamm. 2009;35(4):452.

[27] Aguilar Soto N, Johnson M, Madsen PT, Tyack PL, Bocconcelli A, Fabrizio Borsani J. Does intense ship noise disrupt foraging in deep-diving Cuvier's beaked whales (*Ziphius cavirostris*)? Mar Mamm Sci. 2006;22(3):690–9.

[28] New LF, Moretti DJ, Hooker SK, Costa DP, Simmons SE. Using energetic models to investigate the survival and reproduction of beaked whales (family *Ziphiidae*). PLoS ONE. 2013;8(7):e68725 doi: 10.1371/journal.pone.0068725 23874737 10.1371/journal.pone.0068725 PMC3714291

[29] Hooker SK, Baird RW, Fahlman A. Could beaked whales get the bends?: Effect of diving behaviour and physiology on modelled gas exchange for three species: *Ziphius cavirostris*, *Mesoplodon densirostris* and *Hyperoodon ampullatus*. Respir Physiol Neurobiol. 2009;167(3):235–46. doi: 10.1016/j.resp.2009.04.023 19427415 10.1016/j.resp.2009.04.023

[30] Zimmer WMX, Tyack PL. Repetitive shallow dives pose decompression risk in deep-diving beaked whales. Mar Mamm Sci. 2007;23(4):888–925. doi: 10.1111/j.1748-7692.2007.00152.x

[31] Deruiter SL, Southall BL, Calambokidis J, Zimmer WM, Sadykova D, Falcone EA, et al First direct measurements of behavioural responses by Cuvier's beaked whales to mid-frequency active sonar. Biol Lett. 2013;9(4):1–5. Epub 2013/07/05. doi: 10.1098/rsbl.2013.0223 ; PubMed Central PMCID: PMCPMC3730631. 23825085 10.1098/rsbl.2013.0223 PMC3730631

[32] Tyack PL, Zimmer WM, Moretti D, Southall BL, Claridge DE, Durban JW, et al Beaked whales respond to simulated and actual navy sonar. PLoS ONE. 2011;6(3):e17009 doi: 10.1371/journal.pone.0017009 21423729 10.1371/journal.pone.0017009 PMC3056662

[33] O'Cadhla O, Mackey M, Aguilar de Soto N, Rogan E, Connolly N. Cetaceans and Seabirds of Ireland's Atlantic margin. Volume II—Cetacean Distribution & Abundance. 2004.

[34] Berrow S. Biological diversity of cetaceans (whales, dolphins and porpoises) in Irish waters. Marine Biodiversity in Ireland and Adjacent Waters 2001 p. 115–9.

[35] Evans P. Status Review of Cetaceans in British and Irish Waters Report to UK Department of Environment. Sea Watch Foundation Oxford; 1992.

[36] Evans P, Harding S, Tyler G, Hall S. Analysis of cetacean sightings in the British Isles, 1958–1985: Nature Conservancy Council; 1986.

[37] Rosen M, Evans P, Boran J, Bell G, Thomas C. Cetacean studies in the Celtic Sea, English Channel, and SW North Sea: using training surveys for data collection. European research on cetaceans—13 2000:383–6.

[38] Aguilar de Soto N, Rogan EO, Cadhal O, Gordon JCD, Mackey M, Connolly N. Cetaceans and seabirds of Ireland's Atlantic margin. Volume III—Acoustic surveys for cetaceans. 2004.

[39] O'Brien J, Berrow S, McGrath D, Evans P, editors. Cetaceans in Irish waters: A review of recent research. Biology and Environment: Proceedings of the Royal Irish Academy; 2009: JSTOR.

[40] Bruton T, Cotton D, Enright M. Gulf stream beaked whale *Mesoplodon europaeus* (Gervais). The Irish Naturalists' Journal. 1989;23(4):156–.

[41] Law R, Allchin C, Jones B, Jepson P, Baker J, Spurrier C. Metals and organochlorines in tissues of a Blainville's beaked whale *(Mesoplodon densirostris*) and a killer whale (*Orcinus orca*) stranded in the United Kingdom. Mar Pollut Bull. 1997;34(3):208–12.

[42] Gibson D, Harris E, Bray R, Jepson P, Kuiken T, Baker J, et al A survey of the helminth parasites of cetaceans stranded on the coast of England and Wales during the period 1990–1994. J Zool. 1998;244(4):563–74.

[43] Couzens D, Swash A, Still R, Dunn J. Britain's Mammals: A Field Guide to the Mammals of Britain and Ireland: Princeton University Press; 2017.

[44] Government of Ireland. Wlidlife Act, (1976).

[45] European Commission. The Habitats Directive, © European Union, 1995–2017 (1992).

[46] McCauley RD. Offshore Irish noise logger program (March to September 2014): Analysis of cetacean presence, and ambient and anthropogenic noise sources. Centre for Marine Science and Technology (CMST), Curtin University For RPS MetOcean / Woodside Energy (Ireland) Pty Ltd, 2015.

[47] Wall D, Murray C, O’Brien J, Kavanagh L, Wilson C, Ryan C, et al Atlas of the distribution and relative abundance of the marine mammals in Irish offshore waters 2005–2011. Merchants Quay, Kilrush, Co Clare: Irish Whale and Dolphin Group, 2013.

[48] Edds-Walton PL. Acoustic communication signals of mysticetes whales. Bioacoustics. 1997;8:47–60.

[49] Tyack PL, Clark CW. Communication and acoustic behavior of dolphins and whales In: Au WWL, Popper AN, Fay RR, editors. Hearing by Whales and Dolphins. New York: Springer-Verlag; 2000 p. 156–224.

[50] Wahlberg M, Beedholm K, Heerfordt A, Møhl B. Characteristics of biosonar signals from the northern bottlenose whale, *Hyperoodon ampullatus*. J Acoust Soc Am. 2012;130(5):3077–84. 10.1121/1.3641434 22087935

[51] Hooker SK, Baird RW. Deep–diving behaviour of the northern bottlenose whale, *Hyperoodon ampullatus* (Cetacea: Ziphiidae). Proceedings of the Royal Society of London B: Biological Sciences. 1999;266(1420):671–6. doi: 10.1098/rspb.1999.0688

[52] Martin B, Moors-Murphy H, editors. Analysis of Northern bottlenose whale pulses and associated reflections recorded from the Gully Marine Protected Area. Proceedings of Meetings on Acoustics; 2013: Acoustical Society of America.

[53] Baumann-Pickering S, McDonald MA, Simonis AE, Berga AS, Merkens KP, Oleson EM, et al Species-specific beaked whale echolocation signals. J Acoust Soc Am. 2013;134(3):2293–301. doi: 10.1121/1.4817832 23967959 10.1121/1.4817832

[54] Zimmer WM, Johnson MP, Madsen PT, Tyack PL. Echolocation clicks of free-ranging Cuvier’s beaked whales (*Ziphius cavirostris*). J Acoust Soc Am. 2005;117(6):3919–27. 16018493 10.1121/1.1910225

[55] Cholewiak D, Baumann-Pickering S, Van Parijs S. Description of sounds associated with Sowerby's beaked whales (*Mesoplodon bidens*) in the western North Atlantic Ocean. J Acoust Soc Am. 2013;134(5):3905–12. doi: 10.1121/1.4823843 24180799 10.1121/1.4823843

[56] Gillespie D, Dunn C, Gordon J, Claridge D, Embling C, Boyd I. Field recordings of Gervais' beaked whales *Mesoplodon europaeus* from the Bahamas. J Acoust Soc Am. 2009;125(5):3428–33. doi: 10.1121/1.3110832 PubMed PMID: ISI:000265884700063. 19425681 10.1121/1.3110832

[57] Powers DMW. Evaluation: From precision, recall and F-measure to ROC, informedness, markedness & correlation. Journal of Machine Learning Technologies. 2011;2(1):37–63.

[58] Davis J, Goadrich M, editors. The relationship between Precision-Recall and ROC curves. Proceedings of the 23rd international conference on machine learning; 2006; Pittsburgh, PA: ACM.

[59] Roch MA, Brandes TS, Patel B, Barkley Y, Baumann-Pickering S, Soldevilla MS. Automated extraction of odontocete whistle contours. J Acoust Soc Am. 2011;130(4):2212–23. doi: 10.1121/1.3624821 21973376 10.1121/1.3624821

[60] Martin B. Computing cumulative sound exposure levels from anthropogenic sources in large data sets. Proceedings of Meetings on Acoustics. 2013;19(1):9 http://dx.doi.org/10.1121/1.4800967.

[61] Zar JH. Biostatistical analysis: Pearson Education India; 1999.

[62] Reda I, Andreas A. Solar position algorithm for solar radiation applications. Technical Report. 1617 Cole Boulevard Golden, Colorado 80401–3393 National Renewable Energy Laboratory, Laboratory USDoE; 2004 Revised January 2008 Report No.: NREL/TP-560-34302 Contract No.: 5.

[63] Wiggins SM, Oleson EM, McDonald MA, Hildebrand JA. Blue whale (Balaenoptera musculus) diel call patterns offshore of Southern California. Aquat Mamm. 2005;31(2):161.

[64] Munger LM, Wiggins SM, Moore SE, Hildebrand JA. North Pacific right whale (*Eubalaena japonica*) seasonal and diel calling patterns from long‐term acoustic recordings in the southeastern Bering Sea, 2000–2006. Mar Mamm Sci. 2008;24(4):795–814.

[65] Finneran JJ. Auditory weighting functions and TTS/PTS exposure functions for marine mammals exposed to underwater noise. Technical Report, 2016 May 2016. Report No.

[66] [NMFS] National Marine Fisheries Service. Technical Guidance for Assessing the Effects of Anthropogenic Sound on Marine Mammal Hearing: Underwater Acoustic Thresholds for Onset of Permanent and Temporary Threshold Shifts. U.S. Department of Commerce, NOAA. NOAA Technical Memorandum NMFS-OPR-55; 2016. p. 178.

[67] Zimmer WMX, Harwood J, Tyack PL, Johnson MP, Madsen PT. Passive acoustic detection of deep-diving beaked whales. J Acoust Soc Am. 2008;124(5):2823–32. doi: 10.1121/1.2988277 PubMed PMID: ISI:000260836700018. 19045770 10.1121/1.2988277

[68] Ward J, Morrissey R, Moretti D, DiMarzio N, Jarvis S, Johnson M, et al Passive Acoustic Detection and Localization of *Mesoplodon densirostris* (Blainville's Beaked Whale) Vocalization Using Distributed Bottom-Mounted Hydrophones in Conjunction With a Digital Tag (DTAG) Recording. Naval Undersea Warfare Center Div Newport Ri, 2008.

[69] Wenz GM. Acoustic ambient noise in the ocean: Spectra and sources. J Acoust Soc Am. 1962;34(12):1936–56. PubMed PMID: 2492.

[70] McGovern B, Culloch RM, O'Connell M, Berrow S. Temporal and spatial trends in stranding records of cetaceans on the Irish coast, 2002–2014. J Mar Biol Assoc UK. 2016:1–13.

[71] Hammond P, Macleod K, Gillespie D, Swift R, Winship A, Burt M, et al Cetacean offshore distribution and abundance in the European Atlantic (CODA). Final Report University of Saint Andrews, Scotland 2009.

[72] Santos M, Pierce GJ, Herman J, Lopez A, Guerra A, Mente E, et al Feeding ecology of Cuvier's beaked whale (*Ziphius cavirostris*): a review with new information on the diet of this species. J Mar Biol Assoc UK. 2001;81(4):687–94.

[73] MacLeod CD, Santos M, Pierce GJ. Review of data on diets of beaked whales: evidence of niche separation and geographic segregation. J Mar Biol Assoc UK. 2003;83(3):651–65.

[74] Santos BM, Pierce GJ. A note on niche overlap in teuthophagous whales in the Northern Northeast Atlantic. Phuket Marine Biological Centre Research Bulletin. 2005;66:291–98.

[75] Hastie L, Pierce G, Wang J, Bruno I, Moreno A, Piatkowski U, et al Cephalopods in the north-eastern Atlantic: species, biogeography, ecology, exploitation and conservation. Oceanogr Mar Biol Annu Rev. 2009;47:111–90.

[76] Collins MA, Yau C, Boyle PR, Friese D, Piatkowski U. Distribution of cephalopods from plankton surveys around the British Isles. Bull Mar Sci. 2002;71(1):239–54.

[77] Collins MA, Yau C, Allcock L, Thurston MH. Distribution of deep-water benthic and bentho–pelagic cephalopods from the north-east Atlantic. J Mar Biol Assoc UK. 2001;81(1):105–17.

[78] Clarke MR. Oceanic cephalopod distribution and species diversity in the eastern north Atlantic. 2006.

[79] Lu C, Clarke M. Vertical distribution of cephalopods at 40 N, 53 N and 60 N at 20 W in the North Atlantic. J Mar Biol Assoc UK. 1975;55(1):143–63.

[80] Au WWL, Giorli G, Chen J, Copeland A, Lammers M, Richlen M, et al Nighttime foraging by deep diving echolocating odontocetes off the Hawaiian islands of Kauai and Ni'ihau as determined by passive acoustic monitors. J Acoust Soc Am. 2013;133(5):3119–27. doi: 10.1121/1.4798360 23654414 10.1121/1.4798360

[81] Johnston D, McDonald M, Polovina J, Domokos R, Wiggins S, Hildebrand J. Temporal patterns in the acoustic signals of beaked whales at Cross Seamount. Biol Lett. 2008;4(2):208–11. doi: 10.1098/rsbl.2007.0614 18252660 10.1098/rsbl.2007.0614 PMC2429936

[82] MacLeod CD, Pierce GJ, Santos MB. Geographic and temporal variations in strandings of beaked whales (Ziphiidae) on the coasts of the UK and the Republic of Ireland from 1800–2002. J Cetacean Res Manage. 2004;6(1):79–86.

[83] Vines J, Woodcock R. Marine Mammal Observer Report During Ruadhan 3d Seismic Survey, Porcupine Basin, Licence Blocks 35/13, 35/14, 35/15p, 35/18 & 35/19 Offshore Ireland For Capricorn Ireland Ltd 10th August to 11th September 2014. RPS Energy, 2014.

[84] Lynn P, Mars K. Breanann Survey Marine Mammal Observation Daily Report, 21 July 2016. IWDG Consulting, 2016.

[85] Martín V, Tejedor M, Pérez-Gil M, Dalebout ML, Arbelo M, Fernández A. A Sowerby's Beaked Whale (*Mesoplodon bidens*) Stranded in the Canary Islands: The Most Southern Record in the Eastern North Atlantic. Aquat Mamm. 2011;37(4):512.

[86] Spitz J, Cherel Y, Bertin S, Kiszka J, Dewez A, Ridoux V. Prey preferences among the community of deep-diving odontocetes from the Bay of Biscay, Northeast Atlantic. Deep Sea Research Part I: Oceanographic Research Papers. 2011;58(3):273–82.

[87] Pereira JN, Neves VC, Prieto R, Silva M, Cascão I, Oliveira C, et al Diet of mid-Atlantic Sowerby's beaked whales *Mesoplondon bidens*. Deep Sea Research Part I: Oceanographic Research Papers. 2011;58(11):1084–90.

[88] Rogan E, Hernández-Milián G. Preliminary analysis of beaked whale strandings in Ireland: 1800–2009. International Whaling Commission, IWC SC/63/SM19. 2011.

[89] Johnsen E, Godø O. Diel variations in acoustic recordings of blue whiting (*Micromesistius poutassou*). ICES J Mar Sci. 2007;64(6):1202–9.

[90] Nash R. The diel behaviour of small demersal fish on soft sediments on the west coast of Scotland using a variety of techniques: with special reference to Lesueurigobius friesii (Pisces; Gobiidae). Mar Ecol. 1982;3(2):161–78.

[91] De Pontual H, Jolivet A, Bertignac M, Fablet R. Diel vertical migration of European hake *Merluccius merluccius* and associated temperature histories: insights from a pilot data‐storage tagging (DST) experiment. J Fish Biol. 2012;81(2):728–34. doi: 10.1111/j.1095-8649.2012.03345.x 22803732 10.1111/j.1095-8649.2012.03345.x

[92] Mackey M. SEA678 Data Report for Offshore Cetacean Populations. 2011.

[93] Benjaminsen T, Christensen I. The natural history of the bottlenose whale, *Hyperoodon ampullatus* (Forster) Behav Mar Anim: Springer; 1979 p. 143–64.

[94] Fernández R, Pierce GJ, MacLeod CD, Brownlow A, Reid RJ, Rogan E, et al Strandings of northern bottlenose whales, *Hyperoodon ampullatus*, in the north-east Atlantic: seasonality and diet. J Mar Biol Assoc UK. 2014;94(6):1109–16.

[95] Aguilar de Soto N, Rogan E, Ó Cadhla O, Gordon JCD, Mackey M, Connolly N. Cetaceans and Seabirds of Ireland’s Atlantic Margin. Volume III–Acoustic surveys for cetaceans. Report on research carried out under the Irish Infrastructure Programme (PIP): Rockall Studies Group (RSG) projects 98/6 and 00/13, Porcupine Studies Group project P00/15 and Offshore Support Group (OSG) project 99/38., 2004.

[96] Whitehead H, Gowans S, Faucher A, Mccarrey SW. Population analysis of northern bottlenose whales in the Gully, Nova Scotia. Mar Mamm Sci. 1997;13(2):173–85.

[97] Wimmer T, Whitehead H. Movements and distribution of northern bottlenose whales, *Hyperoodon ampullatus*, on the Scotian Slope and in adjacent waters. Can J Zool. 2004;82(11):1782–94.

[98] Martin SB, Matthews M-NR, MacDonnell JT, Bröker K. Characteristics of seismic survey pulses and the ambient soundscape in Baffin Bay and Melville Bay, West Greenland. J Acoust Soc Am. 2017;142(6):3331–46. doi: 10.1121/1.5014049 29289080 10.1121/1.5014049

